# The Dynamic Brain: From Spiking Neurons to Neural Masses and Cortical Fields

**DOI:** 10.1371/journal.pcbi.1000092

**Published:** 2008-08-29

**Authors:** Gustavo Deco, Viktor K. Jirsa, Peter A. Robinson, Michael Breakspear, Karl Friston

**Affiliations:** 1 Institució Catalana de Recerca i Estudis Avançats (ICREA), Universitat Pompeu Fabra, Department of Technology, Computational Neuroscience, Barcelona, Spain; 2 Theoretical Neuroscience Group, Institut Sciences de Mouvement, Marseille, France; 3 Center for Complex Systems and Brain Sciences, Department of Physics, Florida Atlantic University, Boca, Florida, United States of America; 4 School of Physics, University of Sydney, Sydney, New South Wales, Australia; 5 Brain Dynamics Center, Westmead Millennium Institute, Westmead Hospital and University of Sydney, Westmead, New South Wales, Australia; 6 Faculty of Medicine, University of Sydney, Sydney, New South Wales, Australia; 7 School of Psychiatry, University of New South Wales, Sydney, and The Black Dog Institute, Randwick, New South Wales, Australia; 8 School of Physics, University of Sydney, Sydney, New South Wales, Australia; 9 Wellcome Trust Centre for Neuroimaging, University College London, London, United Kingdom; Indiana University, United States of America

## Abstract

The cortex is a complex system, characterized by its dynamics and architecture, which underlie many functions such as action, perception, learning, language, and cognition. Its structural architecture has been studied for more than a hundred years; however, its dynamics have been addressed much less thoroughly. In this paper, we review and integrate, in a unifying framework, a variety of computational approaches that have been used to characterize the dynamics of the cortex, as evidenced at different levels of measurement. Computational models at different space–time scales help us understand the fundamental mechanisms that underpin neural processes and relate these processes to neuroscience data. Modeling at the single neuron level is necessary because this is the level at which information is exchanged between the computing elements of the brain; the neurons. Mesoscopic models tell us how neural elements interact to yield emergent behavior at the level of microcolumns and cortical columns. Macroscopic models can inform us about whole brain dynamics and interactions between large-scale neural systems such as cortical regions, the thalamus, and brain stem. Each level of description relates uniquely to neuroscience data, from single-unit recordings, through local field potentials to functional magnetic resonance imaging (fMRI), electroencephalogram (EEG), and magnetoencephalogram (MEG). Models of the cortex can establish which types of large-scale neuronal networks can perform computations and characterize their emergent properties. Mean-field and related formulations of dynamics also play an essential and complementary role as forward models that can be inverted given empirical data. This makes dynamic models critical in integrating theory and experiments. We argue that elaborating principled and informed models is a prerequisite for grounding empirical neuroscience in a cogent theoretical framework, commensurate with the achievements in the physical sciences.

## Introduction

The brain appears to adhere to two fundamental principles of functional organization, functional integration and functional specialization, where the integration within and among specialized areas is mediated by connections among them. The distinction relates to that between localisationism and connectionism that dominated thinking about cortical function in the nineteenth century. Since the early anatomic theories of Gall, the identification of a particular brain region with a specific function has become a central theme in neuroscience. In this paper, we address how distributed and specialized neuronal responses are realized in terms of microscopic brain dynamics; we do this by showing how neuronal systems, with many degrees of freedom, can be reduced to lower dimensional systems that exhibit adaptive behaviors.

It is commonly accepted that the information processing underlying brain functions, like sensory, motor, and cognitive functions, is carried out by large groups of interconnected neurons [1]–[4]. Neurons are the cells responsible for encoding, transmitting, and integrating signals originating inside or outside the nervous system. The transmission of information within and between neurons involves changes in the so-called resting membrane potential, the electrical potential of the neurons at rest, when compared to the extracellular space. The inputs one neuron receives at the synapses from other neurons cause transient changes in its resting membrane potential, called postsynaptic potentials. These changes in potential are mediated by the flux of ions between the intracellular and extracellular space. The flux of ions is made possible through ion channels present in the membrane. The ion channels open or close depending on the membrane potential and on substances released by the neurons, namely neurotransmitters, which bind to receptors on the cell's membrane and hyperpolarize or depolarize the cell. When the postsynaptic potential reaches a threshold, the neuron produces an impulse. The impulses or spikes, called action potentials, are characterized by a certain amplitude and duration and are the units of information transmission at the interneuronal level. Information is thought to be encoded in terms of the frequency of the action potentials, called spiking or firing rate (i.e., rate coding), as well as in the timing of action potentials (i.e., temporal coding).

One way to investigate the biological basis of information processing in the brain is to study the response of neurons to stimulation. This can be done in experimental animals using implanted electrodes to record the rates and timing of action potentials. However, this invasive approach is generally not possible in humans. To study brain function in humans, techniques allowing the indirect study of neuronal activity have been developed. An example is functional magnetic resonance imaging (fMRI), measuring regional changes in metabolism and blood flow associated with changes in brain activity. This approach to measuring regional differences in brain activity is possible because at a macroscopic level the cortex is organized into spatially segregated regions known to have functionally specialized roles. A technique such as fMRI allows the mapping of brain regions associated with a particular task or task component.

Understanding the fundamental principles underlying higher brain functions requires the integration of different levels of experimental investigation in cognitive neuroscience (from single neurons, neuroanatomy, neurophysiology, and neuroimaging, to neuropsychology and behavior) via a unifying theoretical framework that captures the neural dynamics inherent in the elaboration of cognitive processes. In this paper, we review and integrate a variety of computational approaches that have been used to characterize the dynamics of the cortex, as evidenced at different levels of measurement.

The paper is structured as follows. The central theme of this review is that the activity in populations of neurons can be understood by reducing the degrees of freedom from many to few, hence resolving an otherwise intractable computational problem. The most striking achievement in this regard is the reduction of a large population of spiking neurons to a distribution function describing their probabilistic evolution—that is, a function that captures the likely distribution of neuronal states at a given time. In turn, this can be further reduced to a single variable describing the mean firing rate. This reduction is covered first, in the next section. In the section entitled Neural Modes and Masses, we return to the full probability distribution function and show how it can be represented by a set of scalars that parameterize it parsimoniously. These parameters are equivalent to the moments of the distribution. In many instances, a few—possibly even one (equivalent to the center of mass)—are sufficient to summarize activity. These are known as Neural Mass Models. These models capture the dynamics of a neuronal population. Naturally, it is useful to understand how neuronal activity unfolds on the spatially continuous cortical sheet. This can be addressed with neural field models; involving differential operators with both temporal and spatial terms. That is, neuronal activity depends on its current state as well as spatial gradients, which allow its spread horizontally across the cortical surface. These models are covered in the Neural Field Models section. In Numerical Simulations: Ensemble Activity from Neuronal to Whole Brain Scale, we provide numerical simulations of neuronal ensemble dynamics across a hierarchy of spatial and temporal scales. At the microscopic scale, we simulate an entire array of spiking neurons in response to a sensory-evoked synaptic current. By comparing the response to that of a mesoscopic neural mass model, we show what is gained and what is lost by abstracting to a more tractable set of evolution equations. The spread of activity across the cortical surface, in a neural field model, is also illustrated. Finally, in the section entitled Cognitive and Clinical Applications, we illustrate applications of neural ensemble modeling in health and disease; namely, decision-making, auditory scene analysis, and absence seizures.

A summary of the notation for all the main dynamical variables and physiological parameters is given in Table 1.

**Table 1:** List of notation and symbols.

Quantity	Symbol	SI Unit
Neural membrane potential	*V*	V
Neural membrane capacitive current	*I*	A
Time elapsed since most recent action potential	*T*	s
Neural membrane capacitance	*C*	F
Neural membrane resistance	*R*	*ω*
Neural membrane time constant	*τ* = *RC*	s
Neural leak or resting potential	*V_L_*	V
Neural firing threshold	*θ*	V
Neural action potential spike	*δ*	
Neural refractory period	*T_ref_*	s
Neural reset (post-firing) membrane potential	*V_reset_*	V
Synaptic efficacy of cell *j* onto cell *i*	*J_ij_*	A s^−1^
Neural membrane phase space state vector	*ν*	varies
Neural ensemble probability density	*p*(*ν*,*t*)	varies
Neural ensemble dynamics (flow)	*f*(*ν*,*t*)	varies
Neural ensemble random fluctuations (dispersion)	*D*(*ν*,*t*)	varies
Neural ensemble jacobian	*Q*	varies
Neural ensemble average synaptic efficacy	〈*J*〉*_J_*	A s^−1^
Neural ensemble mean firing rate	*Q*(*t*)	s^−1^
Neural ensemble infinitesimal depolarization	*ε* = *dV*(*t*)	V
Neural ensemble mean membrane potential (drift)	*μ* = * ˙μ_ν_*	V
Neural ensemble mean capacitive current	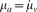	A
Neural ensemble membrane potential variance (diffusion)	*σ^2^*	(V)^2^
Neural ensemble probability density flux	*F*	varies
Neural population transfer function	*φ*(*ν*,*σ*)	s^−1^
Neural population probability basis functions (modes)	*η*(*ν*)	varies
Neural mass synaptic gain time constant	*κ*	s^−1^
Neural mass synaptic response coefficient	*γ*	dimensionless
Neural field local membrane potential in population *a*	*V_a_*(*r*,*t*)	V
Neural field local firing rate in population *a*	*Q_a_*(*r*,*t*)	s^−1^
Mean number of synapses on neuron *a* from neurons *b*	*N_ab_*	
Mean time-integrated strength of the response of *V_a_* per incoming spike from neurons *b*	*s_ab_*	*μ* V s
Average rate of incoming spikes (pulse density) between populations *a* and *b*	*φ_ab_*	s^−1^
Discrete time delay between populations *a* and *b*	*τ_ab_*	s
Coupling strength between neural populations *a* and *b*	*ν_ab_* = *N_ab_s_ab_*	V s
Mean decay rate of the soma response to a delta-function synaptic input	*α_ab_*	s^−1^
Mean rise rate of the soma response to a delta-function synaptic input	*β_ab_*	s^−1^
Firing threshold for channels of type *a*	*θ_a_*	V
Characteristic range of axons, including dendritic arborization	*r*, *r_ab_*	m
Characteristic action potential propagation velocity	*c*, *c_ab_*	m s^−1^
Temporal damping coefficient in the absence of pulse regeneration	*γ_a_* = *c_a_/r_a_*	s^−1^
Steady state sigmoid slope in population *a*	*ρ_a_*	(V s)^−1^
Macroscopic observable	*ψ*(*k*,*ω*) = **M** ***Q***	
Spatiotemporal measurement matrix	**M**	
Autonomous (uncoupled) growth/decay rate of neural mass	*ζ*	s^−1^

The first column gives a brief description of the parameter, with its symbol listed in the second. The unit of each quantity is given in the third column.

## Mean-Field Models

This section provides an overview of mean-field models of neuronal dynamics and their derivation from models of spiking neurons. These models have a long history spanning a half-century (e.g., [5]) and are formulated using concepts from statistical physics. In this section, we try to clarify some key concepts and show how they relate to each other. Models are essential for neuroscience, in the sense that the most interesting questions pertain to neuronal mechanisms and processes that are not directly observable. This means that questions about neuronal function are generally addressed by inference on models or their parameters, where the model links neuronal processes that are hidden from our direct observation. Broadly speaking, models are used to generate data, to study emergent behaviors, or they can be used as forward or observation models, which are inverted given empirical data. This inversion allows one to select the best model (given some data) and make probabilistic comments about the parameters of that model. Mean-field models are suited to data which reflect the behavior of a population of neurons, such as the electroencephalogram (EEG), magnetoencephalogram (MEG), and fMRI. The most prevalent models of neuronal populations or ensembles are based upon something called the mean-field approximation. The mean-field approximation is used extensively in statistical physics and is essentially a technique that finesses an otherwise computationally or analytically intractable problem. An exemplary approach, owing to Boltzmann and Maxwell, is the approximation of the motion of molecules in a gas by mean-field terms such as temperature and pressure.

### 

#### Ensemble density models

Ensemble models attempt to model the dynamics of large (theoretically infinite) populations of neurons. Any single neuron could have a number of attributes; for example, post-synaptic membrane depolarization, *V*, capacitive current, *I*, or the time since the last action potential, *T*. Each attribute induces a dimension in the phase space of a neuron; in our example the phase space would be three dimensional and the state of each neuron would correspond to a point *ν* = {*V*,*I*,*T*} ∈ℜ^3^ or particle in phase space. Imagine a very large number of neurons that populate phase space with a density *p*(*ν*,*t*). As the state of each neuron evolves, the points will flow through phase space, and the ensemble density *p*(*ν*,*t*) will evolve until it reaches some steady state or equilibrium. *p*(*ν*,*t*) is a scalar function returning the probability density at each point in phase space. It is the evolution of the density per se that is characterized in ensemble density methods. These models are particularly attractive because the density dynamics conform to a simple equation: the Fokker-Planck equation
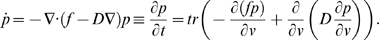
(1)This equation comprises a flow and a dispersion term; these terms embed the assumptions about the dynamics (phase flow, *f*(*ν*,*t*)) and random fluctuations (dispersion, *D*(*ν*,*t*)) that constitute our model at the neuronal level. This level of description is usually framed as a (stochastic) differential equation (Langevin equation) that describes how the states evolve as functions of each other and some random fluctuations with
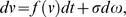
(2)where, *D* = ½*σ* ^2^ and *ω* is a standard Wiener process; i.e., *w*(*t*)−*w*(*t*+Δ*t*)∼*N*(0, Δ*t*). Even if the dynamics of each neuron are complicated, or indeed chaotic, the density dynamics remain simple, linear, and deterministic. In fact, we can write the density dynamics in terms of a linear operator or Jacobian *Q*

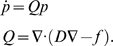
(3)


In summary, for any model of neuronal dynamics, specified as a stochastic differential equation, there is a deterministic linear equation that can be integrated to generate ensemble dynamics. In what follows, we will explain in detail the arguments that take us from the spiking behavior of individual neurons to the mean-field dynamics described by the Fokker-Planck equation. We will consider the relationship between density dynamics and neural mass models and how these can be extended to cover spatiotemporal dynamics in the brain.

#### From spiking neurons to mean-field models

The functional specialization of the brain emerges from the collective network dynamics of cortical circuits. The computational units of these circuits are spiking neurons, which transform a large set of inputs, received from different neurons, into an output spike train that constitutes the output signal of the neuron. This means that the spatiotemporal spike patterns produced by neural circuits convey information among neurons; this is the microscopic level on which the brain's representations and computations rest [6]. We assume that the nonstationary temporal evolution of the spiking dynamics can be captured by one-compartment, point-like models of neurons, such as the leaky integrate-and-fire (LIF) model [7] used below. Other models relevant for systems neuroscience can be found in [4],[8],[9]. In the LIF model, each neuron *i* can be fully described in terms of a single internal variable, namely the depolarization *V_i_*(*t*) of the neural membrane. The basic circuit of a LIF model consists of a capacitor, *C*, in parallel with a resistor, *R*, driven by a synaptic current (excitatory or inhibitory postsynaptic potential, EPSP or IPSP, respectively). When the voltage across the capacitor reaches a threshold *θ*, the circuit is shunted (reset) and a *δ* pulse (spike) is generated and transmitted to other neurons. The subthreshold membrane potential of each neuron evolves according to a simple *RC* circuit, with a time constant *τ* = *RC* given by the following equation:
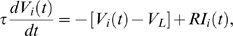
(4)where *I_i_*(*t*) is the total synaptic current flow into the cell *i* and *V_L_* is the leak or resting potential of the cell in the absence of external afferent inputs. In order to simplify the analysis, we neglect the dynamics of the afferent neurons (see [10] for extensions considering detailed synaptic dynamics such as AMPA, NMDA, and GABA). The total synaptic current coming into the cell *i* is therefore given by the sum of the contributions of *δ*-spikes produced at presynaptic neurons. Let us assume that *N* neurons synapse onto cell *i* and that *J_ij_* is the efficacy of synapse *j*, then the total synaptic afferent current is given by
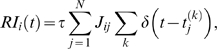
(5)where 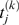 is the emission time of the *k^th^* spike from the *j^th^* presynaptic neuron. The subthreshold dynamical Equation 4, given the input current (from Equation 5), can be integrated, and yields
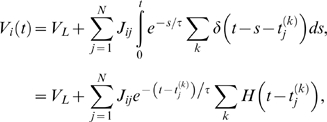
(6,7)if the neuron *i* is initially (*t* = 0) at the resting potential (*V_i_*(0) = *V_L_*). In Equation 7, *H*(*t*) is the Heaviside function (*H*(*t*) = 1 if *t*>0, and *H*(*t*) = 0 if *t*<0). Thus, the incoming presynaptic *δ*-pulse from other neurons is basically low-pass filtered to produce an EPSP or IPSP in the post-synaptic cell. Nevertheless, the integrate-and-fire (IF) model is not only defined by the subthreshold dynamics but includes a reset after each spike generation, which makes the whole dynamics highly nonlinear. In what follows, we present a theoretical framework which is capable of dealing with this.

#### The population density approach

Realistic neuronal networks comprise a large number of neurons (e.g., a cortical column has O(10^4^)−O(10^8^) neurons) which are massively interconnected (on average, a neuron makes contact with O(10^4^) other neurons). The underlying dynamics of such networks can be described explicitly by the set of coupled differential equations (Equation 4) above. Direct simulations of these equations yield a complex spatiotemporal pattern, covering the individual trajectory of the internal state of each neuron in the network. This type of direct simulation is computationally expensive, making it very difficult to analyze how the underlying connectivity relates to various dynamics. In fact, most key features of brain operation seem to emerge from the interplay of the components; rather than being generated by each component individually. One way to overcome these difficulties is by adopting the population density approach, using the Fokker-Planck formalism (e.g., [11]). As noted above, the Fokker-Planck equation summarizes the flow and dispersion of states over phase space in a way that is a natural summary of population dynamics in genetics (e.g., [12]) and neurobiology (e.g., [13],[14]).

In what follows, we derive the Fokker-Planck equation for neuronal dynamics that are specified in terms of spiking neurons. This derivation is a little dense but illustrates the approximating assumptions and level of detail that can be captured by density dynamics. The approach we focus on was introduced by [15] (see also [16],[17]). In this approach, individual IF neurons are grouped together into populations of statistically similar neurons. A statistical description of each population is given by a probability density function that expresses the distribution of neuronal states (i.e., membrane potential) over the population. In general, neurons with the same state *V*(*t*) at a given time *t* have a different history because of random fluctuations in the input current *I*(*t*). The main source of randomness is from fluctuations in recurrent currents (resulting from “quenched” randomness in the connectivity and transmission delays) and fluctuations in the external currents. The key assumption in the population density approach is that the afferent input currents impinging on neurons in one population are uncorrelated. Thus, neurons sharing the same state *V*(*t*) in a population are indistinguishable. Consequently, the dynamics are described by the evolution of the probability density function:
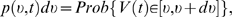
(8)which expresses the population density, which is the fraction of neurons at time *t* that have a membrane potential *V*(*t*) in the interval [*ν*,*ν*+*dν*]. The evolution of the population density is given by the Chapman-Kolmogorov equation
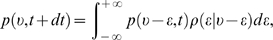
(9)where *ρ*(*ε*|*ν*) = *Prob*{*V*(*t*+*dt*) = *ν*+*ε*|*V*(*t*) = *ν*} is the conditional probability that generates an infinitesimal change *ε* = *V*(*t*+*dt*)−*V*(*t*) in the infinitesimal interval *dt*. The Chapman-Kolmogorov equation can be written in a differential form by performing a Taylor expansion in *p*(*ν*′,*t*) *ρ*(*ε*|*ν*′) around *ν*′ = *ν*; i.e.,
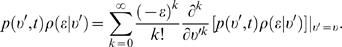
(10)In the derivation of the last equation, we have assumed that *p*(*ν*′,*t*) and *ρ*(*ε*| *ν*′) are infinitely many times differentiable in *ν*. Inserting this expansion in Equation 9, and replacing the time derivative in *ν*′ by the equivalent time derivative in *ν*, we obtain
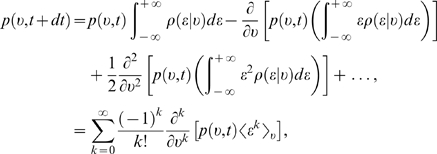
(11,12)where 〈…〉*_ν_* denotes the average with respect to *ρ*(*ε*| *ν*) at a given *ν*. Finally, taking the limit for *dt* → 0, we obtain:
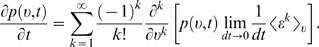
(13)


Equation 13 is known as the Kramers-Moyal expansion of the original integral Chapman-Kolmogorov equation (Equation 9). It expresses the time evolution of the population density in differential form.

#### The diffusion approximation

The temporal evolution of the population density as given by Equation 13 requires the moments 〈*ε^k^*〉*_υ_* due to the afferent current during the interval *dt*. These moments can be calculated by the mean-field approximation. In this approximation, the currents impinging on each neuron in a population have the same statistics, because as we mentioned above, the history of these currents is uncorrelated. The mean-field approximation entails replacing the time-averaged discharge rate of individual cells with a common time-dependent population activity (ensemble average). This assumes ergodicity for all neurons in the population. The mean-field technique allows us to discard the index denoting the identity of any single neuron and express the infinitesimal change, *dV*(*t*), in the membrane potential of all neurons as:
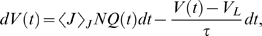
(14)where *N* is the number of neurons, and *Q*(*t*) is the mean population firing rate. This is determined by the proportion of active neurons by counting the number of spikes *n_spikes_*(*t*,*t*+*dt*) in a small time interval *dt* and dividing by *N* and by *dt* [18]; i.e.,
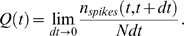
(15)


In Equation 14, 〈*J*〉*_J_* denotes the average of the synaptic weights in the population. The moments of the infinitesimal depolarization, *ε* = *dV*(*t*), can now be calculated easily from Equation 14. The first two moments in the Kramers-Moyal expansion are called drift and diffusion coefficients, respectively, and they are given by:
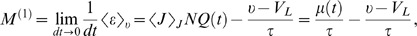
(16)

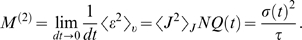
(17)In general, keeping only the leading term linear in *dt*, it is easy to prove that for *k*>1,
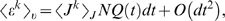
(18)and hence,
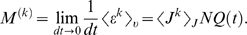
(19)


The diffusion approximation arises when we neglect high-order (*k*>2) terms. The diffusion approximation is exact in the limit of infinitely large networks, i.e., *N* → ∞, if the synaptic efficacies scale appropriately with network size, such that *J* → 0 but *NJ^2^* → *const*. In other words, the diffusion approximation is appropriate, if the minimal kick step, *J*, is very small but the overall firing rate is very large. In this case, all moments higher than two become negligible, in relation to the drift (*μ*) and diffusion (*σ* ^2^) coefficients.

The diffusion approximation allows us to omit all higher orders *k*>2 in the Kramers-Moyal expansion. The resulting differential equation describing the temporal evolution of the population density is called the Fokker-Planck equation, and reads
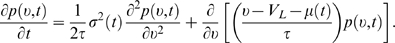
(20)


In the particular case that the drift is linear and the diffusion coefficient, *σ* ^2^(*t*), is given by a constant, the Fokker-Planck equation describes a well-known stochastic process called the Ornstein-Uhlenbeck process [19]. Thus, under the diffusion approximation, the Fokker-Planck equation (Equation 20) expresses an Ornstein-Uhlenbeck process. The Ornstein-Uhlenbeck process describes the temporal evolution of the membrane potential *V*(*t*) when the input afferent currents are given by
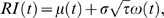
(21)where *ω*(*t*) is a white noise process. Under the diffusion approximation, Equation 21 can also be interpreted (by means of the Central Limit Theorem), as the case in which the sum of many Poisson processes (Equation 5) becomes a normal random variable with mean *μ*(*t*) and variance *σ* ^2^.

#### The mean-field model

The simulation of a network of IF neurons allows one to study the dynamical behavior of the neuronal spiking rates. Alternatively, the integration of the non-stationary solutions of the Fokker-Planck equation (Equation 20) also describes the dynamical behavior of the network, and this would allow the explicit simulation of neuronal and cortical activity (single cells, EEG, fMRI) and behavior (e.g., performance and reaction time). However, these simulations are computationally expensive and their results probabilistic, which makes them unsuitable for systematic explorations of parameter space. However, the stationary solutions of the Fokker-Planck equation (Equation 20) represent the stationary solutions of the original IF neuronal system. This allows one to construct bifurcation diagrams to understand the nonlinear mechanisms underlying equilibrium dynamics. This is an essential role of the mean-field approximation: to simplify analyses through the stationary solutions of the Fokker-Planck equation for a population density under the diffusion approximation (Ornstein-Uhlenbeck process) in a self-consistent form. In what follows, we consider stationary solutions for ensemble dynamics.

The Fokker-Planck equation describing the Ornstein-Uhlenbeck process, with *μ* = 〈*J*〉*_J_ NQ*(*t*) and *σ* ^2^ = 〈*J^2^*〉*_J_ NQ*(*t*), can be rewritten as a continuity equation:
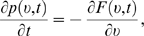
(22)where *F* is the flux of probability defined as follows:
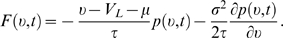
(23)The stationary solution should satisfy the following boundary condition:
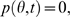
(24)and
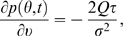
(25)which expresses the fact that the probability current at threshold gives, by a self-consistent arguments, the average firing rate, *Q*, of the population. Furthermore, at *ν*→−4 the probability density vanishes fast enough to be integrable; i.e.,
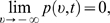
(26)and
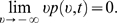
(27)In addition, the probability mass leaving the threshold at time *t* has to be re-injected at the reset potential at time *t*+*t_ref_* (where *t_ref_* is the refractory period of the neurons), which can be accommodated by rewriting Equation 22 as follows:
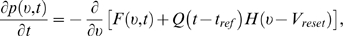
(28)where *H*(.) is the Heaviside function. The solution of Equation 28 satisfying the boundary conditions (Equations 24–27) is:
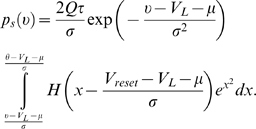
(29)Taking into account the fraction of neurons, *Qt_ref_*, in the refractory period and the normalization of the mass probability,
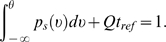
(30)Finally, substituting Equation 29 into Equation 30, and solving for *Q*, we obtain the population transfer function, *φ*, of Ricciardi [13]:
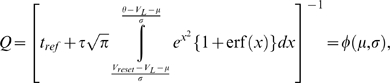
(31)where 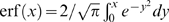.

The stationary dynamics of each population can be described by the population transfer function, which provides the average population rate as a function of the average input current. This can be generalized easily for more than one population. The network is partitioned into populations of neurons whose input currents share the same statistical properties and fire spikes independently at the same rate. The set of stationary, self-reproducing rates, *Q_i_*, for different populations, *i*, in the network can be found by solving a set of coupled self-consistency equations, given by:
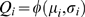
(32)


To solve the equations defined by Equation 32 for all *i*, we integrate the differential equation below, describing the approximate dynamics of the system, which has fixed-point solutions corresponding to Equation 32:
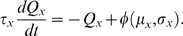
(33)


This enables a posteriori selection of parameters, which induce the emergent behavior that we are looking for. One can then perform full nonstationary simulations using these parameters in the full IF scheme to generate true dynamics. The mean-field approach ensures that these dynamics will converge to a stationary attractor that is consistent with the steady-state dynamics we require [10],[20]. In our case, the derived transfer function, *φ*, corresponds consistently to the assumptions of the simple LIF model described in the From Spiking-Neurons to Mean-Field Models section. Further extension for more complex and realistic models are possible. For example, an extended mean-field framework, which is consistent with the IF and realistic synaptic equations that considers both the fast and slow glutamatergic excitatory synaptic dynamics (AMPA and NMDA) and the dynamics of GABA inhibitory synapses, can be found in [10]. Before turning to neural mass models, we consider some applications of mean-field modeling that will be reprised in the last section.

#### Competition and cooperation

How are different cortical representations integrated to form a coherent stream of perception, cognition, and action? The brain is characterized by a massive recurrent connectivity between cortical areas, which suggests that integration of partial representations might be mediated by cross talk via interareal connections. Based on this view [21], and neurophysiological evidence [22], it has been hypothesized that each cortical area represents a set of alternative hypotheses, encoded in the activities of cell assemblies. Representations of conflicting hypotheses compete with each other; however, each area represents only a part of the environment or internal state. In order to arrive at a coherent global representation, different cortical areas bias each others' internal representations by communicating their current states to other areas, thereby favoring certain sets of local hypotheses over others. By recurrently biasing each others' competitive internal dynamics, the neocortical system arrives at a global representation in which each area's state is maximally consistent with those of the other areas. This view has been referred to as the biased-competition hypothesis. In addition to this competition-centered view, a cooperation-centered picture of brain dynamics, where global representations find their neural correlate in assemblies of coactivated neurons, has been formulated [21],[23]. Coactivation is achieved by increased connectivity among the members of each assembly. Reverberatory communication between the members of the assembly then leads to persistent activation to engender temporally extended representations.

The mean-field approach has been applied to biased-competition and cooperation networks and has been used to model single neuronal responses, fMRI activation patterns, psychophysical measurements, effects of pharmacological agents, and effects of local cortical lesions [6], [24]–[33]. In the section entitled Cognitive and Clinical Applications, we present one of these examples, in the context of decision-making.

## Neural Modes and Masses

The Fokker-Planck equation, (Equation 1), is a rather beautiful and simple expression that prescribes the evolution of ensemble dynamics, given any initial conditions and equations of motion that embed our neuronal model. However, it does not specify how to encode or parameterize the density itself. There are several approaches to this. These include binning the phase space and using a discrete approximation to a continuous density. However, this can lead to a vast number of differential equations, especially if there are multiple states for each population. One solution to this is to reduce the number of states (i.e., dimension of the phase space) to render the integration of the Fokker-Planck more tractable. One elegant example of this reduction can be found in [34]. Here, population dynamics are described by a set of one-dimensional partial differential equations in terms of the distributions of the refractory density (where the refractory state is defined by the time elapsed since the last action potential). This furnishes realistic simulations of the population activity of hippocampal pyramidal neurons, based on something known as the refractory density equation and a single-neuron threshold model. The threshold model is a conductance-based model with adaptation-providing currents.

An alternative approach to dimension reduction is to approximate the ensemble densities with a linear superposition of probabilistic modes or basis functions *η*(*ν*) that cover phase space. In this section, we overview this modal approach to ensemble dynamics, initially in the general setting and then in the specific case, where the dynamics can be captured by the activity of a single node.

### 

#### Moments and modes of density dynamics

Instead of characterising the density dynamics explicitly, one can summarize it in terms of coefficients parameterising the expression of modes:
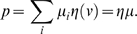
(34)where *μ* = *η* ^−^ *p*, *η* ^−^ being the generalized inverse of the matrix encoding the basis set of modes.

A useful choice for the basis functions are the eigenfunctions (i.e., eigen vectors) of the Fokker-Planck operator, *Q* [17], where *Qη* = *ηλ*⇒*η* ^−^ *Qη* = *λ* and *λ* is a leading-diagonal matrix of eigenvalues. Because the Fokker-Planck operator conserves probability mass, all its real eigenvalues are zero or negative. In the absence of mean-field effects, the biorthogonality of the eigenfunctions effectively uncouples the dynamics of the modes they represent
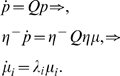
(35)


The last expression means that, following perturbation, each mode decays exponentially, to disclose the equilibrium mode, *η*_0_, that has a zero eigenvalue. Because the eigenvalues are complex (due to the fact that the Jacobian is not symmetric), the decay is oscillatory in nature, with a frequency that is proportional to the imaginary part of the eigenvalue and a rate constant proportional to the real part. The key thing about this parameterisation is that most modes will decay or dissipate very quickly. This means we only have to consider a small number of modes, whose temporal evaluation can be evaluated simply with
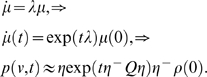
(36)


See [35] for an example of this approach, in which the ensuing nonlinear differential equations were used in a forward model of observed data. In summary, we can formulate the ensemble dynamics of any neuronal system, given its equations of motion, using the equation above. This specifies how the coefficients of probability modes would evolve from any initial state or following a perturbation to the neuronal states. It furnishes a set of coupled differential equations that can be integrated to form predictions of real data or to generate emergent behaviors. We have introduced parameterisation in terms of probability modes because it provides a graceful link to neural mass models.

#### Neural mass models

Neural mass models can be regarded as a special case of ensemble density models, where we summarize our description of the ensemble density with a single number. Early examples can be found in the work of [5],[36],[37]. The term mass action model was coined by [38] as an alternative to density dynamics. These models can be motivated as a description in terms of the expected values of neuronal states, *μ*, under the assumption that the equilibrium density has a point mass (i.e., a delta function). This is one perspective on why these simple mean-field models are called neural mass models. In short, we replace the full ensemble density with a mass at a particular point and then summarize the density dynamics by the location of that mass. What we are left with is a set of nonlinear differential equations describing the evolution of this mode. But what have we thrown away? In the full nonlinear Fokker-Planck formulation, different phase functions or probability density moments could couple to each other; both within and between populations or ensembles. For example, this means that the average depolarisation in one ensemble could be affected by the dispersion or variance of depolarisation in another. In neural mass models, we ignore this possibility because we can only couple the expectations or first moments. There are several devices that are used to compensate for this simplification. Perhaps the most ubiquitous is the use of a sigmoid function, *ς*(*μ_ν_*), relating expected depolarisation to expected firing rate [38]. This implicitly encodes variability in the postsynaptic depolarisation, relative to the potential at which the neuron would fire. A common form for neural mass equations of motion posits a second order differential equation for expected voltage, or, equivalently, two coupled first order equations, *μ* = {*μ_ν_*,*μ_i_*} where
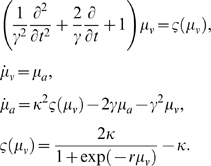
(37)Here *μ_a_* can be regarded as capacitive current. The constant *γ* controls the rise time of voltage, in response to inputs (see also the Neural Field Models section). These differential equations can be expressed as a convolution of inputs, *ς*(*μ_ν_*), to give the expected depolarization, *μ_ν_*; i.e., the convolution of the input signal with an impulse response kernel *W*(*t*)
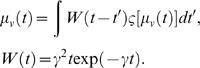
(38)


The input is commonly construed to be a firing rate (or pulse density) and is a sigmoid function, *ς*, of mean voltage of the same or another ensemble. The coupling constant, *κ*, scales the amplitude of this mean-field effect. This form of neural mass model has been used extensively to model electrophysiological recordings (e.g., [39]–[41]) and has been used recently as the basis of a generative model for event-related potentials that can be inverted using real data [42].

In summary, neural mass models are special cases of ensemble density models that are furnished by ignoring all but the expectation or mean of the ensemble density. This affords a considerable simplification of the dynamics and allows one to focus on the behavior of a large number of ensembles, without having to worry about an explosion in the number of dimensions or differential equations one has to integrate. The final sort of model we will consider is the generalisation of neural mass models that allow for states that are functionals of position on the cortical sheet. These are referred to as neural field models and are discussed in the following sections.

## Neural Field Models

The density dynamics and neural mass models above covered state the attributes of point processes, such as EEG sources, neurons, or neuronal compartments. An important extension of these models speaks to the fact that neuronal dynamics play out on a spatially extended cortical sheet. In other words, states like the depolarisation of an excitatory ensemble in the granular layer of cortex can be regarded as a continuum or field, which is a function of space, *x*, and time, *μ*(*t*)→*μ*(*x*,*t*). This allows one to formulate the dynamics of the expected field in terms of partial differential equations in space and time. These are essentially wave equations that accommodate lateral interactions. Although we consider neural field models last, they were among the first mean-field models of neuronal dynamics [43],[44]. Key forms for neural field equations were proposed and analysed by [45]–[47]. These models were generalized by [48],[49] who, critically, considered delays in the propagation of spikes over space. The introduction of propagation delays leads to dynamics that are very reminiscent of those observed empirically.

Typically, neural field models can be construed as a spatiotemporal convolution (c.f., Equation 38) that can be written in terms of a Green's function; e.g.,
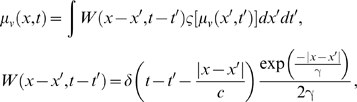
(39)where |*x*−*x′*| is the distance between the spatial locations *x* and *x′*, *c* is the characteristic speed of spike propagation, and *γ* reflects the spatial decay of lateral interactions. The corresponding second order equations of motion are a neural wave equation (see [48],[49] and below)
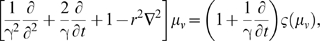
(40)where *γ* = *c*/*r* and ▽^2^ is the Laplacian. The formal similarity with the neural mass model in (37) is self-evident. These sorts of models have been extremely useful in modeling spatiotemporally extended dynamics (e.g., [50]–[53]). The generic form of neural field dynamics can be written as (see also [53]):
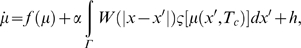
(41)where *μ* = *μ*(*x*,*t*) is the neural field, capturing the neural mass activity at time *t* and position *x*. *f*(*μ*) captures the local dynamics of the neural field, and *T_c_* = *t*−|*x*−*x′*|/*c* is the time delay due to signal propagation. *h* is a constant threshold value and Γ is the spatial domain of the neural field, where *x* ∈ Γ = [0,*L*]. The kernel *W*(|*x*−*x′*|) denotes the connectivity function, which is translationally invariant in space, i.e., the probability that two neural masses are connected depends only on the distance between them. If we neglect the local dynamics *f*(*μ*), 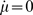, and use an exponential kernel as in Equation 39, we recover Equations 39 and 40. This approximation is valid when the axonal delays contribute mostly to the dynamics, for instance in large-scale networks, when the local dynamics are much faster than the network dynamics. It is easy to show that most realistic connectivity kernels provide a neural wave equation like Equation 40; this is due to the fact that the connectivity must remain integrable. As above, the parameter *c* is the propagation velocity of action potentials traveling down an axon. If *f*(*μ*) = −*ζ μ*, then the constant *ζ*>0∈ℜ represents the growth rate of the neural mass. *α*>0 is a scaling constant. Under instantaneous interactions, *c*→∞, single population models with locally excitatory and laterally inhibitory connectivity can support global periodic stationary patterns in one dimension as well as single or multiple localized solutions (bumps and multi-bumps) [47]. This class of models are also sometimes referred to as continuous attractor neural networks (CANN). When the firing rate, *ς*, is a Heaviside step function, [45] was able to construct an explicit one-bump solution of the form
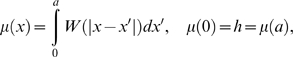
(42)where the value *a* corresponds to the width of the bump. Amari also identified criteria to determine if only one bump, multiple bumps, or periodic solutions exist and if they are stable. This simple mathematical model can be extended naturally to accommodate multiple populations and cortical sheets, spike frequency adaptation, neuromodulation, slow ionic currents, and more sophisticated forms of synaptic and dendritic processing as described in the review articles [4],[54],[55]. Spatially localized bump solutions are equivalent to persistent activity and have been linked to working memory in prefrontal cortex [56],[57]. During behavioral tasks, this persistent elevated neuronal firing can last for tens of seconds after the stimulus is no longer present. Such persistent activity appears to maintain a representation of the stimulus until the response task is completed. Local recurrent circuitry has received the most attention, but other theoretical mechanisms for the maintenance of persistent activity, including local recurrent synaptic feedback and intrinsic cellular bistability [58],[59], have been put forward. The latter will be captured by specific choices of the local dynamics, *f*(*μ*), in Equation 41; for instance, [60] choose a cubic-shaped function of the firing rate, which, under appropriate parameters, allows for intrinsic bistability. Single bump solutions have been used for neural modeling of the head-direction system [61]–[64], place cells [65]–[68], movement initiation [69], and feature selectivity in visual cortex, where bump formation is related to the tuning of a particular neuron's response [70]. Here the neural fields maintain the firing of its neurons to represent any location along a continuous physical dimension such as head direction, spatial location, or spatial view. The mathematical analysis of the neural field models is typically performed with linear stability theory, weakly nonlinear perturbation analysis, and numerical simulations. With more than one population, nonstationary (traveling) patterns are also possible. In two dimensions, many other interesting patterns can occur, such as spiral waves [71], target waves, and doubly periodic patterns. These latter patterns take the form of stripes and checkerboard-like patterns, and have been linked to drug-induced visual hallucinations [72]. For smooth sigmoidal firing rates, no closed-form spatially localized solutions are known, though much insight into the form of multibump solutions has been obtained using techniques first developed for the study of fourth-order pattern forming systems [73]. Moreover, in systems with mixed (excitatory and inhibitory) connectivity or excitatory systems with adaptive currents, solitary traveling pulses are also possible. The bifurcation structure of traveling waves in neural fields can be analysed using a so-called Evans function and has recently been explored in great detail [74].

Much experimental evidence, supporting the existence of neural fields, has been accumulated (see [53] for a summary). Most of these results are furnished by slice studies of pharmacologically treated tissue, taken from the cortex [75]–[77], hippocampus [78], and thalamus [79]. In brain slices, these waves can take the form of synchronous discharges, as seen during epileptic seizures [80], and spreading excitation associated with sensory processing [81]. For traveling waves, the propagation speed depends on the threshold, *h*, which has been established indirectly in real neural tissue (rat cortical slices bathed in the GABA-A blocker picrotoxin) by [82]. These experiments exploit the fact that (i) cortical neurons have long apical dendrites and are easily polarized by an electric field, and (ii) that epileptiform bursts can be initiated by stimulation. A positive (negative) electric field applied across the slice increased (decreased) the speed of wave propagation, consistent with the theoretical predictions of neural field theory, assuming that a positive (negative) electric field reduces (increases) the threshold, *h*, in Equation 42.

### 

#### Recent developments in neural field models

More and more physiological constraints have been incorporated into neural field models of the type discussed here (see Equations 39 and 40). These include features such as separate excitatory and inhibitory neural populations (pyramidal cells and interneurons), nonlinear neural responses, synaptic, dendritic, cell-body, and axonal dynamics, and corticothalamic feedback [38], [43], [44], [48], [50], [83]–[87]. A key feature of recent models is that they use parameters that are of functional significance for EEG generation and other aspects of brain function; for example, synaptic time constants, amount of neurotransmitter release or reuptake, and the speed of signal propagation along dendrites. Inferences can also be made about the parameters of the nonlinear IF response at the cell body, and about speeds, ranges, and time delays of subsequent axonal propagation, both within the cortex and on extracortical paths (e.g., via the thalamus). It is also possible to estimate quantities that parametrize volume conduction in tissues overlying the cortex, which affect EEG measurements [88], or hemodynamic responses that determine the blood oxygen level–dependent (BOLD) signals [89]. Each of these parameters is constrained by physiological and anatomical measurements, or, in a few cases, by other types of modeling. A key aim in modeling is to strike a balance between having too few parameters to be realistic, and too many for the data to be able to constrain them effectively.

Recent work in this area has resulted in numerous quantitatively verified predictions about brain electrical activity, including EEG time series [86],[87],[90], spectra [50],[86],[87],[90],[91], coherence and correlations, evoked response potentials (ERPs) [87], and seizure dynamics [86],[90],[92]. Inversion of these models has also furnished estimates of underlying physiological parameters and their variations across the brain, in different states of arousal and pathophysiology [86],[93],[94].

There are several interesting aspects to these modeling initiatives, which generalize the variants discussed in earlier sections: (i) synaptic and dendritic dynamics and summation of synaptic inputs to determine potentials at the cell body (soma), (ii) generation of pulses at the axonal hillock, and (iii) propagation of pulses within and between neural populations. We now look more closely at these key issues.

#### *Synaptodendritic dynamics and the soma potential*

Assume that the brain contains multiple populations of neurons, indexed by the subscript *a*, which labels simultaneously the structure in which a given population lies (e.g., a particular nucleus) and the type of neuron (e.g., interneuron, pyramidal cell). Then the spatially continuous soma potential, *V_a_*, is the sum of contributions, *V_ab_*, arriving as a result of activity at each type of (mainly) dendritic synapse *b*, where *b* indexes both the incoming neural population and the neurotransmitter type of the receptor. (Note that *V_a_* is linearly related to the current reaching the soma, and to *μ* in earlier sections.) Thus we write
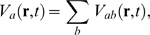
(43)where **r** = (*x*,*y*) denotes the spatial coordinates, *t* the time. The summation is assumed to be linear, and all potentials are measured relative to the resting potential [95]. For moderate perturbations relative to a steady state, the value of the resting potential can be subsumed into the values of other parameters [95]. As above, the cortex is approximated as a 2-D sheet and **r** is assumed to be the actual position in the case of the cortex; other structures, such as the thalamus, are linked to the cortex via a primary topographic map. This map links points in a one-to-one manner between structures; i.e., we assign the same value of r to such points. Hence, in structures other than the cortex, this dimensional map coordinate, **r**, denotes a rescaled physical dimension (i.e. the physical coordinate multiplied by the ratio of the cortical scale to the structure's scale), a point that should be remembered when interpreting values of spatial parameters in these structures.

The subpotentials, *V_ab_*, respond in different ways to incoming spikes, depending on their synaptic dynamics (ion-channel kinetics, diffusion in the synaptic cleft, etc.), and on subsequent signal dispersion in the dendrites. The resulting soma response to a delta-function input at the synapse can be approximated via the differential equation [50].
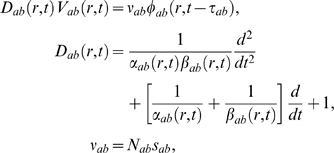
(44,45,46)where *ν_ab_* is a coupling strength, *N_ab_* is the mean number of synapses on neuron *a* from neurons *b*, *s_ab_* is the mean time-integrated strength of the response of *V* per incoming spike, and *θ_ab_* is the average rate of incoming spikes (allowing for the possibility of a discrete time delay, *τ_ab_*, between populations *b* and *a* in addition to any delays due to spreading within populations). The parameter *α_ab_* is the mean decay rate of the soma response to a delta-function synaptic input, while *β_ab_* is the mean rise rate: this biexponential form has been found to be a good approximation [8],[50],[96],[97]. If the *α_ab_* and *β_ab_* are independent of *b* (which is not generally the case), then the subscript *b* on *D_ab_* can be omitted and *V_a_* itself satisfies Equation 44 with the right side of Equation 44 replaced by the sum of *P_ab_* over *b*. This approximation is also valid if *α* and *β* are interpreted as effective values, averaged over subpopulations.

#### *Pulse generation*

In cells with voltage-gated ion channels, action potentials are produced at the axonal hillock when the soma potential exceeds some threshold *θ_a_*. When averaged over a population of neurons, with normal response characteristics, a reasonable approximation for the firing rate, *Q*, is
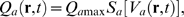
(47)where *Q_a_*_max_ is the maximum firing rate and *S_a_* is a monotonic increasing sigmoidal function that approaches zero as *V_a_*→−∞ and unity as *V_a_*→∞. A commonly used approximation is
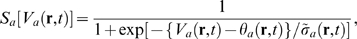
(48)where *θ_a_* is the firing threshold for channels of type *a* and 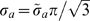 is the standard deviation of the threshold over the population.

#### *Axonal propagation*

Spatiotemporal propagation of pulses within and between populations determines the values of *φ_ab_*. If we indicate the firing rate *Q_a_* for the cell type *a* by a subscript, then *φ_ab_* can be expressed in terms of the firing rate at other locations and earlier times. If we assume linear propagation, signals propagate as described by the neural field equation (Equation 40).
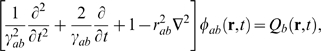
(49)where, as per Equation 40, *r_ab_* is the characteristic range of axons, including dendritic arborization, *c_ab_* is the characteristic velocity of signals in these axons, and *γ_ab_* = *c_ab_* / *r_ab_* is the resulting temporal damping coefficient in the absence of pulse regeneration. Note that, in comparision to the use of the terms *μ_ν_* and *ς*(*μ_ν_*) in Equation 40, the present wave-equation is formalized in relationship to population-specific pulse densities, *φ_ab_*, and firing rates, *Q_a,b_*. By employing population-specific fields and parameters, it allows each population to generate a family of outgoing fields that propagate to different populations in different ways. Equation 49 is also satisfied if *φ_ab_* is replaced by the free propagator 
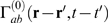
 and the right side is replaced by a source of the form *δ*(**r**−**r′**)*δ*(*t*−*t′*). In Fourier space, this gives
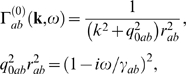
(50,51)where ***k*** = (k_x_,k_y_) is the wave vector and *ω* is the angular frequency. Critically, the neural field Equation 49 enables very diffuse (i.e., not topographically specific) connections between populations to be handled straightforwardly, simply by increasing *r_ab_* while reducing *γ_ab_*, thereby allowing influences to propagate long distances with little damping.

#### *Parameters and modulations*

The above equations contain a number of parameters encoding physiology and anatomy (e.g., coupling strengths, firing thresholds, time delays, velocities, etc.). In general, these can vary in space, due to differences among brain regions, and in time, due to effects like habituation, facilitation, and adaptation. In brief, time-dependent effects can be included in neural field models by adding dynamical equations for the evolution of the parameters. Typically, these take a form in which parameter changes are driven by firing rates or voltages, with appropriate time constants. The simplest such formulation is [95]

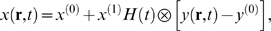
(52)where *x* is the evolving parameter, *y* is the quantity that drives the evolution, *x* ^(0)^ and *y* ^(0)^ are steady state values, and *x* ^(1)^ is a constant that describes the strength of feedback. The symbol ⊗ indicates a convolution of the driver with the temporal response function *H*(*t*), which incorporates the time constants of the dynamics. If we use the normalized form
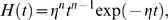
(53)then we find the differential equivalent of Equation 53:
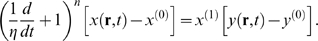
(54)


#### *Steady states and dynamics*

Here, we first discuss how to find the steady states of neural field models. Important phenomena have been studied by linearizing these models around their steady state solutions. Hence, we discuss linear properties of such models, including how to make predictions of observable quantities from them; including transfer functions, spectra, and correlation and coherence functions. In doing this, we assume for simplicity that all the model parameters are constant in time and space, although it is possible to relax this assumption at some cost in complexity. Linear predictions from neural field models have accounted successfully for a range of experimental phenomena, as mentioned above. Nonlinear dynamics of such models have also been discussed in the literature, resulting in successful predictions of epileptic dynamics, for example [86],[92], but are not considered here (but see the Cognitive and Clinical Applications section).

*Steady states and global dynamics*. Previous work has shown that many properties of neuronal dynamics can be obtained by regarding activity changes as perturbations of a steady state [86]. Spatially uniform steady states can be obtained by solving the preceding equations with all time and space derivatives set to zero, assuming that the parameters are spatially constant. The spatially uniform steady states are thus the solutions of the set of equations
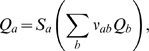
(55)which are generally transcendental in form.

*Linear equations for activity*. Of the relevant equations above, all but Equation 48 are linear in *Q*. Equation 48 can be linearized by replacing the sigmoid, *S_a_*, by its slope, *ρ_a_*, at the steady state value of *V_a_*; we also approximate this quantity as constant. If we Fourier transform the resulting set of linear equations, we find for the fluctuating parts
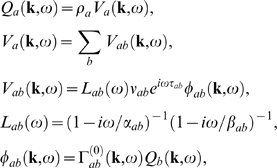
(56,57,58,59,60)where 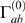 is given by Equation 50 and we have assumed that all the parameters of the equations (but not the fields of activity) are constant on the timescales of interest. Note that we have assumed the system to be unbounded in order to employ a continuous Fourier transform here. The case of bounded systems with discrete spatial eigenmodes can be treated analogously.

For any given spatial wavenumber, **k**, and temporal frequency, *ω*, Equations 56–60 can be rearranged to obtain
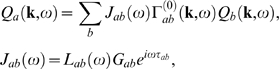
(61,62)where the gains are defined by *G_ab_* = *ρ_a_ν_ab_*.

If there are *N*′ neural populations and *J*′ stimulus sources, and we assume that there is no feedback of stimuli on themselves, or of the brain on stimuli, then we can write Equation 61 as
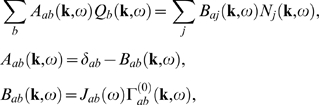
(63,64,65)where the sum on the left of Equation 63 extends only over populations in the brain, while the sum on the right covers only stimulus sources, denoted by *j*. *Q_j_* is written as *N_j_* to make the distinction between population firing rates and incoming stimulus rates absolutely clear. We can now write Equation 63 in matrix form as
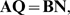
(66)where **A** is an *N*′×*N*′ matrix, ***Q*** is an *N*′-element column vector, **B** is an *N*′×*J*′ matrix, and **N** is a *J*′-element column vector. We can simply invert Equation 66 to find ***Q*** in terms of the stimuli **N**:
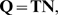
(67)where **T** = **A** ^−1^ **B** is the *N*′×*J*′ transfer matrix of the system. The element *T_aj_* is the response of *Q_a_* to a change in *N_j_* at the same frequency and wave vector.

*Observables*. A measurable scalar quantity *ψ*, such as an EEG scalp voltage or voltage difference, can generally be approximated by a linear function of the firing rates, *Q_a_*. For example, a scalp potential may involve contributions from several populations, with various weights (that may include filtering by volume conduction effects). In this case, at given *ω*,
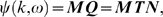
(68)where **M** is an *N*′-element row vector of complex-valued measurement coefficients that encode spatiotemporal filtering characteristics, phase shifts, etc. For example, the coefficients of the matrix **M** can be chosen such that *ψ*(**k**,*ω*) = *φ_ab_*(**k**,*ω*). Further classes of measurement functions are those relating the neural activity to, for example, local field potentials, multiunit activity, the blood oxygen level–dependent (BOLD) response that forms the basis of functional magnetic resonance imaging (fMRI), the metabolic responses underlying positron emission tomography (PET), or single-photon emission computed tomography (SPECT). In what follows, we will implicitly absorb **M** into **T** for simplicity.

*Dispersion and stability*. The dispersion relation of linear waves in the system is given by
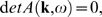
(69)and the system is stable at a particular real **k** if all the frequency roots of this equation have negative imaginary parts. If the steady state is stable for all **k**, spectra and other properties of the linear perturbations can be self-consistently defined; otherwise a fully nonlinear analysis is needed.

*Spectra*. The power spectral density of *ψ* at **k** and *ω* is
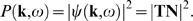
(70)The frequency and wavenumber spectra are then
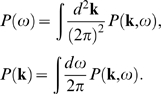
(71,72)A position-dependent frequency cross-spectrum can be calculated from Equation 70:
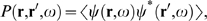
(73)where the angle brackets denote an average over multiple trials and/or over the phase of the exogenous stimuli that drive the system. The spectrum at a particular point, **r**, is *P*(**r**,**r**′,*ω*).

*Correlation and coherence functions*. In steady state, the two-point correlation function can be obtained from Equation 73 via the Wiener-Khinchtine theorem, giving
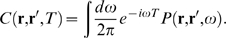
(74)In the case where the system is statistically uniform, Equation 74 depends only on the separation **R** = **r**′−**r**, giving
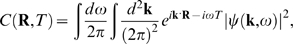
(75)where
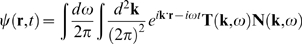
(76)has been used and the arguments of **T** and **N** have been shown for emphasis. At **R** = 0, Equation 74 becomes the Fourier transform of the local power spectrum. In terms of the above expressions, the normalized correlation function and the coherence function, which are both used widely in the literature, are
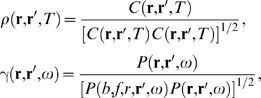
(77,78)respectively.

*Time series and evoked potentials*. The time series of *ψ* at a given point can be obtained via the transfer function, by first calculating the Fourier form of the stimuli that generate it; these can be background noise sources, discrete impulses, or sinusoidal drives, for example. In the case of an impulsive stimulus, the resulting ERP is obtained by setting
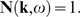
(79)


Similarly, for a spatially uniform sinusoidal drive, the resulting steady state evoked potential (SSEP) is obtained by using
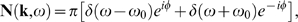
(80)where *ω*_0_ is the drive frequency and *φ* is its phase.

*Case of one long-range population*. An important case, in many applications, is the situation where spatial spreading of activity is dominated by the axons of one population, typically because they have the longest range, are most numerous, or have the highest axonal velocity. In this case, one can ignore the **k** dependence in the other propagators, and it becomes possible to express the transfer function with elements of the form
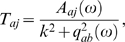
(81)where 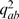 is typically a complicated expression depending on the various *J_ab_*(*ω*).

#### *Heterogeneous connectivity in neural fields*

The brain's network dynamics depend on the connectivity within individual areas, as well as generic and specific patterns of connectivity among cortical and subcortical areas [4],[9],[98]. Intrinsic or intracortical fibers are confined to cortical gray matter in which the cortical neurons reside; these intrinsic connections define the local connectivity within an area. Intracortical fibers are mostly unmyelinated and extend laterally up to 1 cm (in the human brain) with excitatory and inhibitory connections. Their distribution is mostly invariant under spatial translations (homogeneous) [84],[99], which fits the assumptions on the connectivity function in neural fields so far. On the other hand, the corticocortical (extrinsic) fiber system contains fibers which leave the gray matter and connect distant areas (up to 20 cm [84]). This fiber system is myelinated, which increases the transmission speed by an order of magnitude, and is not invariant under spatial translations (heterogeneous); in fact it is patchy [99]. Due to finite transmission speeds, time delays of interareal communication can reach 50–100 ms [84], which is not negligible. Several studies have focused on spatially continuous neural fields, which describe the temporal change of neural activity on local scales, typically within a brain area (see [4],[54],[55] for reviews), assuming homogeneous connectivity and time delays. As discussed in the previous section, early attempts include neural field theories which approximate the large-scale components of the connectivity matrix as translationally invariant and decaying over space [45],[48],[50]. These approaches have been successful in capturing key phenomena of large-scale brain dynamics, including characteristic EEG power spectra [45],[50], epilepsy [92], and MEG activity during sensorimotor coordination [49]. Here we review extensions of these efforts and address network stability under variation of (i) intracortical (intrinsic) connectivity, (ii) transmission speed, and (iii) length of corticocortical (extrinsic) fibers. All three anatomical attributes undergo characteristic changes during the development of the human brain and its function, as well changing in the aged and diseased brain (see [9] for an overview). As a first step, we can split the connectivity function, *W*, into two parts, the homogeneous connectivity, *W_hom_*(|*x*−*y*|), which depends only on the distance, and the heterogeneous connectivity, *W_het_*(*x*,*y*), which captures the effects of the extrinsic fiber system (for an alternative approach with applications to visual gamma phenomena, see [100]–[102]). We can then rewrite the neural field equation as follows:
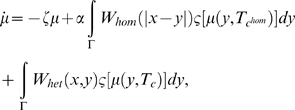
(82)where *T_c_* = *t*−|*x*−*y*| /*c* and *c* is the propagation speed through the heterogeneous corticocortical or extrinsic connections. These fibers are myelinated and hence to be distinguished from the typically unmyelinated (hence slower) intracortical fibers. The latter intrinsic fibers have a transmission speed of *c^hom^* and a transmission delay 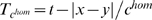. If *W_het_* describes the connectivity of *n* areas, then it can always be written as a sum of two-point connections via
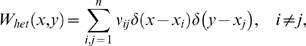
(83)where *ν_ij_* ∈ ℜ again represents the coupling strength between areas at *x_i_* and *x_j_*. The fixed point solution is given by *μ*_0_(*x*) with 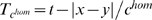. To gain insight into the linear stability of this equilibrium solution *μ*_0_(*x*), we perform a mode expansion of *μ*(*x*,*t*) into a set of spatial basis functions {*ϕ_k_*(*x*)} such that
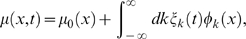
(84)where *ξ_k_*(*t*) is the time-dependent amplitude related to the spatial basis function *ϕ_k_*(*x*). The adjoint set of spatial biorthogonal basis functions is denoted by 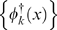. It will be generally true (except in degenerate cases) that only one spatial pattern will become unstable first. For simplicity, we consider the stationary solution *μ*_0_(*x*) = 0 to be the rest state and consider its deviations *μ*(*x*,*t*) = *ξ_k′_*(*t*)*ϕ_k′_* (*x*)+*c*.*c*., where *c*.*c*. denotes the complex conjugate. Then the linear stability of each temporal mode is given by
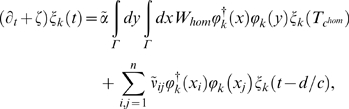
(85)where *d* = |*x_i_*−*x_j_*|>0. Also, 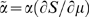 and 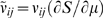 (but for simplicity, we drop the tilde in 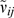 from now on).

Let us pause for a moment and reflect upon the significance of Equation 85. Equation 85 describes the rate of (temporal) change, ∂*_t_ξ_k_*(*t*), of its corresponding spatial neural activation pattern, *ϕ_k_*(*x*). This pattern will change as a function of its own spatial configuration, *ϕ_k_*(*x*), the connections (*W_hom_* and *ν_ij_*), and, last but not least, the transmission times of information exchange, 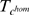 and *d*/*c*. If the rate of change, ∂*_t_ξ_k_*(*t*), is positive, then the particular pattern *ϕ_k_*(*x*) is unstable, otherwise it is stable. In other words, Equation 85 identifies quantitatively how a particular neural activation is impacted by its local and global connectivity in a biologically realistic environment, including signal exchange with finite and varying (intracortical versus corticocortical) transmission speeds. Every treatment of the interplay of anatomical connectivity (local and global connections) and functional connectivity (network dynamics) will have to be represented in the form of Equation 85 or a variation thereof. In this sense, we have here achieved our goal stated in the introduction of this section.

**Figure 1:**
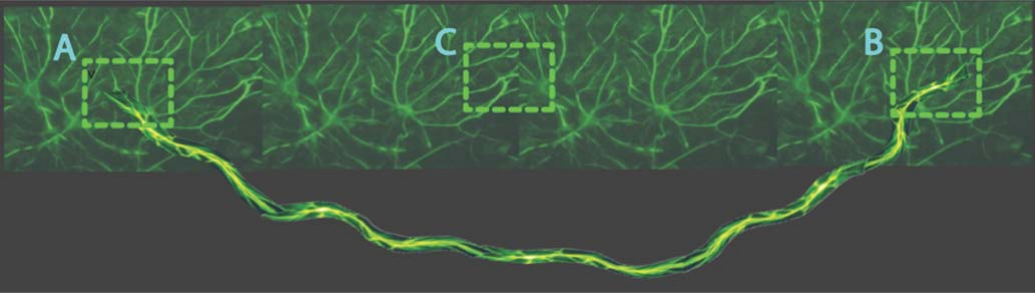
Anatomical connectivity, *W* = *W_hom_*+*W_het_*, comprising homogeneous and heterogeneous connections. The intracortical connections are illustrated as densely connected fibers in the upper sheet and define the homogeneous connectivity *W_hom_*. A single fiber connects the two distant regimes (A) and (B) and contributes to the heterogeneous connectivity, *W_het_*, whereas regime (C) has only homogeneous connections.

To illustrate the effects of interplay between anatomical and functional connectivity, we discuss a simple example following [103],[104]. We assume that there exists only a single corticocortical fiber with terminals at locations *x*_1_ and *x*_2_, that is *n* = 2. Then we have an architecture as shown in Figure 1. Our objective is to identify the stability boundaries of the rest state activity, here the equilibrium solution *μ*_0_(*x*) = 0.

We will consider eigenfunctions of the form *ϕ_k_*(*x*) = *e^ikx^*. Changing the variables such that *z* = *y*−*x* and assuming a solution of the form *ξ_k_*(*t*) = *e^λt^*, *λ* ∈ *C*, the stability condition can then be determined by the following characteristic equation:
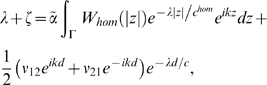
(86)


**Figure 2:**
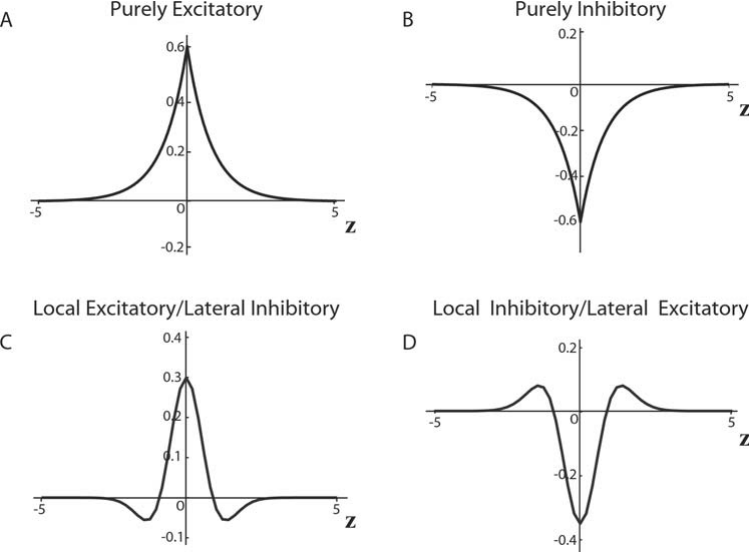
Typical homogeneous connectivity kernels, *W_hom_*(*z*), used for local architectures plotted as a function of spatial distance *z*. Purely excitatory connectivity is plotted in (A); purely inhibitory in (B); center-on, surround-off in (C); and center-off, surround-on in (D). The connectivity kernel in (C) is the most widely used in computational neuroscience.

Linear stability of Equation 86 is obtained if *Re*[*λ*]<0 and is lost, according to [105], at *Re*[*λ*] = 0, that is *λ* = *iω*. Figure 2 shows various connectivity kernels, *W_hom_*, that are often found in the literature.

**Figure 3:**
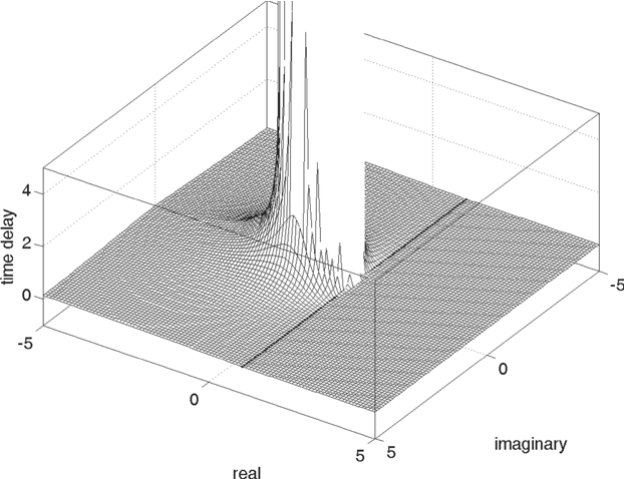
Minimal stable regions for the equilibrium state of a neural field as a function of its connectivity and time delay *τ* = *d*/*c*. The critical surface, at which the equilibrium state undergoes an instability, is plotted as a function of the real and imaginary part of the eigenvalue of its connectivity, *W*. Regimes below the surface indicate stability, above instability. The vertical axis shows the time delay via transmission along the heterogeneous fiber.

Qubbaj and Jirsa [104] discussed the properties of the characteristic Equation 86 in detail, considering separately the special cases of symmetric and asymmetric connectivity, *W*. The characteristic equation defines the critical boundary in the parameter space of *ν*_12_, *ν*_21_, *c*, *c* ^hom^, at which the resting activity, *μ*_0_(*x*) = 0, becomes unstable. Recall that *c* and *c* ^hom^ are the conduction velocities along extrinsic and intrinsic axons, respectively. The general result of [104] can be represented as a critical surface separating stable from unstable regimes as shown in Figure 3. Here the critical transmission delay, *τ* = *d*/*c*, through the heterogeneous fiber is plotted as a function of the real and imaginary part of the eigenvalue of the connectivity, *W*. Essentially a heterogeneous fiber with symmetric weights, *ν*_21_ = *ν*_12_ = *ν*, has only real eigenvalues, whereas asymmetries result in imaginary components. We find that for positive values of *ν* greater than a critical value, the system becomes unstable through a non-oscillatory instability for all values of *c*, *c* ^hom^, (bold line in Figure 3). Within the cylindrical component of the surface, the equilibrium of the system remains always stable for all values of *c*, *c* ^hom^, and hence a time delay shows no effect. In the other regimes of the critical surface, the system typically destabilizes via an oscillatory instability, *ω*≠0, and is sensitive to time delays. The surface shown in Figure 3 represents the lower bound of stability with *c*/*c^hom^* = 1. Increases of the ratio, *c*/*c^hom^*, equivalent to increases of degree of myelination, result in a larger enclosed volume by this surface, i.e., increase of stability. The largest stability region is that for a purely inhibitory kernel followed by that of a local inhibitory and lateral excitatory kernel. The next largest involves a local excitatory and lateral inhibitory kernel. The smallest stability region is obtained for a purely excitatory kernel.

**Figure 4:**
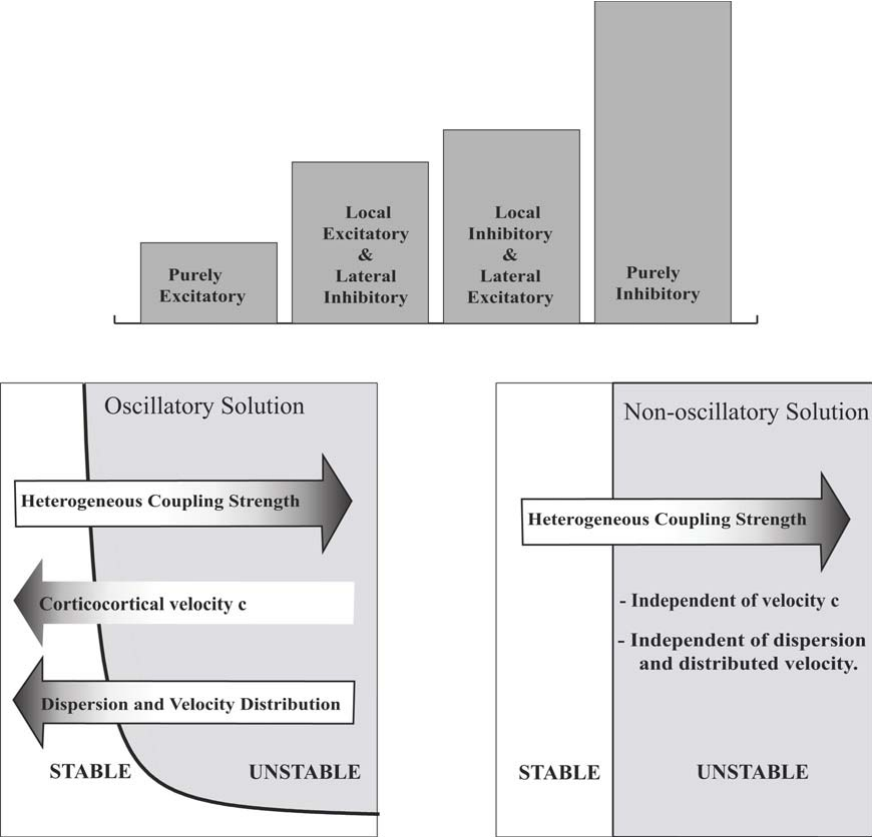
Summary of the stability changes of a neural field with mixed (local/global) connectivity. (Top) The relative size of stability area for different connectivity kernels. (Bottom) Illustration of change of stability as a function of various factors. Gradient within the arrows indicates the increase of the parameter indicated by each arrow. The direction of the arrow refers to the effect of the related factor on the stability change. The bold line separating stable and unstable regions indicates the course of the critical surface as the time delay changes.

A surprising result is that all changes of the extrinsic pathways have the same qualitative effect on the stability of the network, independent of the local intrinsic architecture. This is not trivial, since despite the fact that extrinsic pathways are always excitatory the net effect on the network dynamics could have been inhibitory, if the local architecture is dominated by inhibition. Hence qualitatively different results on the total stability could have been expected. Such is not the case, as we have shown here. Obviously the local architecture has quantitative effects on the overall network stability, but not qualitatively differentiated effects. Purely inhibitory local architectures are most stable, purely excitatory architectures are the least stable. The biologically realistic and interesting architectures, with mixed excitatory and inhibitory contributions, play an intermediate role. When the stability of the network's fixed point solution is lost, this loss may occur through an oscillatory instability or a nonoscillatory solution. The loss of stability for the nonoscillatory solution is never affected by the transmission speeds, a direct physical consequence of its zero frequency allowing time for all parts of the system to evolve in unison. The only route to a non-oscillatory instability is through the increase of the heterogeneous connection strength. For oscillatory instabilities, the situation is completely different. An increase of heterogeneous transmission speeds always causes a stabilization of the global network state. These results are summarized in Figure 4.

## Numerical Simulations: Ensemble Activity from Neuronal to Whole Brain Scales

This section illustrates neuronal ensemble activity at microscopic, mesoscopic, and macroscopic spatial scales through numeric simulations. Our objective is to highlight some of the key notions of ensemble dynamics and to illustrate relationships between dynamics at different spatial scales.

### 

#### Ensemble dynamics at the microscopic scale

To illustrate ensemble dynamics from first principles, we directly simulate a network of coupled neurons which obey deterministic evolution rules and receive both stochastic and deterministic inputs. The system is constructed to embody, at a microscopic level, the response of the olfactory bulb to sensory inputs, as originally formulated by Freeman [38], [106]–[108]. Specifically, in the absence of a sensory input, neurons fire sporadically due to background stochastic inputs. The presence of additional synaptic currents due to a sensory input (e.g., inhaled odor) evokes a bifurcation onto a limit cycle or chaotic attractor. Note that in this section we simulate dynamics at the scale of coupled individual neurons. We can derive directly the predicted ensemble mean response by simply summing over all neurons. We compare this with an explicit model of neural mass dynamics at the mesoscopic scale in the subsequent section.

#### *Microscopic evolution equations*

Each neuron is modeled as a planar reduction of the Hodgkin-Huxley model [109],[110], namely,
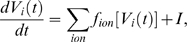
(87)where *f_ion_* introduces conductance-determined transmembrane currents through voltage-dependent channels, *ion* = {*Na^+^*,*K^+^*} and *I* are synaptic currents. The planar reduction has slow potassium channel kinetics but fast sodium channels, whose states vary directly with transmembrane potential [111]. Synaptic currents are modeled, for the present purposes, to arise from three sources,
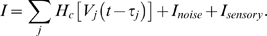
(88)


**Figure 5:**
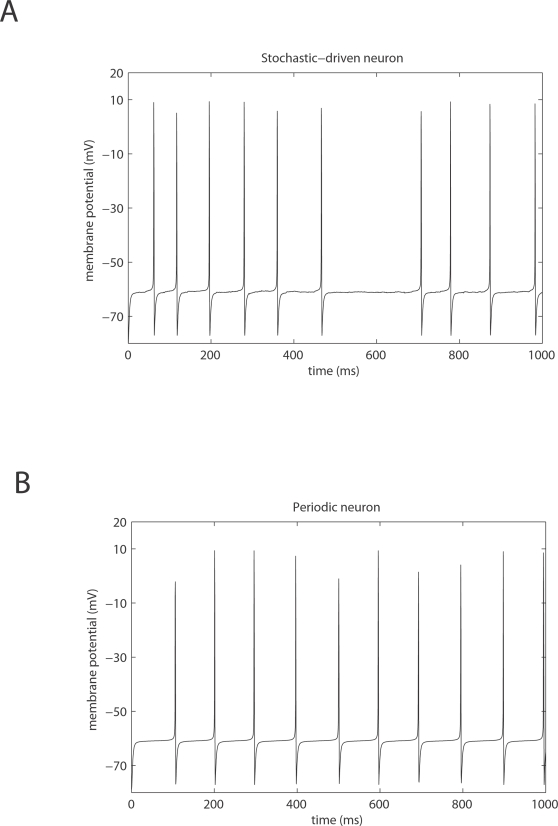
Planar spiking neuron. (A) Stochastically perturbed fixed point. (B) Limit cycle attractor.

The first term represents recurrent feedback from neurons within the ensemble due to their own firing. The coupling term, *H_c_*, incorporates both the nature of the (all-to-all) within-ensemble coupling and the EPSP with parametric strength *c*. For the present purposes, the EPSP consists of a brief steady current whenever the presynaptic neuron is depolarized. The external currents, *I_noise_*, introduce stochastic inputs (e.g., from brain stem inputs) and are modeled as a constant flow with a superimposed Poisson train of discrete pulses. The final term, *I_sensory_*, models sensory input, consisting of a constant synaptic current to a subset of neurons, whenever the sensory stimulus is present. Hence this system permits an exploration of the relative impact of the flow (deterministic) and diffusive (stochastic) effects as embodied at the ensemble level by the Fokker-Planck equation (Equation 20) at the neuronal network level. The Nernst potentials, conductances, and background current are set so that, in the absence of noise and sensory inputs, each neuron rests just below a saddle-node bifurcation to a limit cycle [109]. This implies that neurons are spontaneously at rest (quiescent) but depolarize with a small perturbation. If the perturbation is due to a stochastic train, then the neuron fires randomly at an average rate proportional to the stochastic inputs. However, following a small increase in the constant flow term, due to a sensory input, *I_sensory_*, the quiescent state becomes unstable and the neuron evolves on a (noise-modulated) limit cycle. Figure 5 shows a stochastically driven neuron (A) compared to a noise-modulated periodic neuron (B). In the former case, the activity is dominated by the stochastic terms. In the latter case, the limit cycle dynamics dominate, although the stochastic inputs modulate the depolarization amplitude.

#### *Microscopic dynamics*

**Figure 6:**
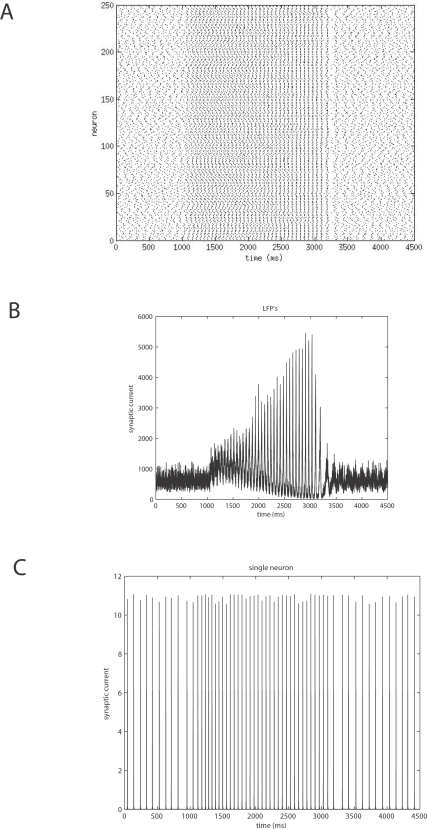
Results of simulating an ensemble of 250 neurons with sensory evoked synaptic currents to all neurons between *t* = 1,000 ms and t = 3,000 ms. (A) Raster plot. (B) Mean synaptic currents. (C) Time series of a single neuron. The effect of the input is to effect a bifurcation in each neuron from stochastic to limit cycle dynamics (phase locking), suppressing the impact of the spatially uncorrelated stochastic inputs. As evident in (A), the increased firing synchrony leads in turn to a marked increase in the simulated local field potentials. The mean synaptic currents evidence an emergent phenomenon, and not merely the superposition of a bursting neuron, as can be seen in (C): clearly no burst is evident at this scale.

Figure 6 shows the results of simulating an ensemble of 250 neurons with a sensory input to all neurons between *t* = 1,000 ms to *t* = 3,000 ms. Figure 6A shows a raster plot of the neural spike timing whilst Figure 6B shows the simulated local field potential from the ensemble ( = total current flow across all neurons). As constructed, the effect of the input is to effect a bifurcation in each neuron from stochastic to limit cycle dynamics. The secondary effect of the appearance of limit cycle dynamics is to suppress the impact of the spatially uncorrelated stochastic inputs. Hence the neurons show an evolution towards phase locking, which was not present prior to the stimulus. As evident in Figure 6B, the increased firing synchrony leads in turn to a marked increase in the simulated local field potentials as individual neurons begin to contribute concurrent ion currents. Once the stimulus ends, there is a brief quiescent phase because all of the neurons have just fired and require a short train of stochastic inputs before they commence firing again. Interestingly, there is evidence of damped mean-field oscillations in the ensemble following stimulus termination, abating after some further 800 ms. To underscore the observation that the mean synaptic currents evidence an emergent phenomenon, and not merely the super-position of a bursting neuron, the time series of a single neuron is provided in Figure 6C. Clearly no burst is evident at this scale.

**Figure 7:**
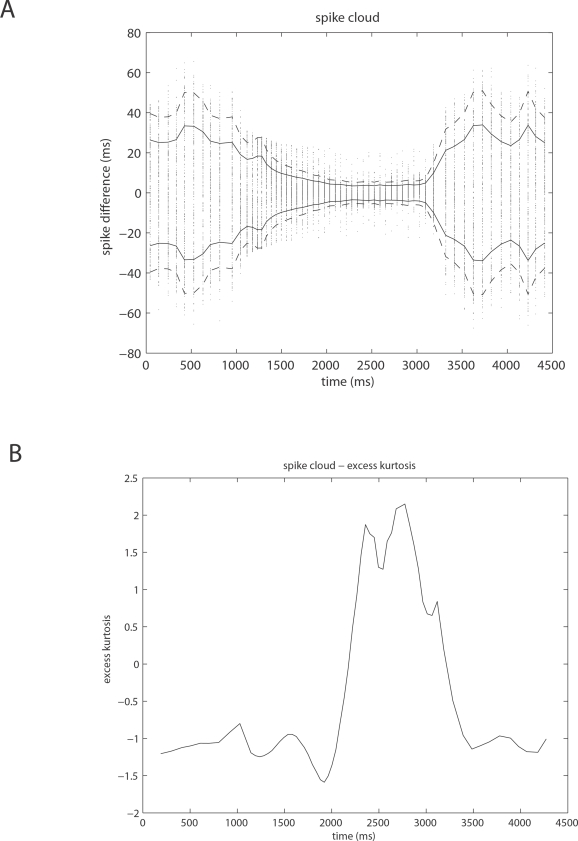
Contraction of spike-timing differences due to synaptic inputs. A seed neuron is chosen at random and the interneuron spike difference for all other neurons is plotted each time it spikes. (A) Solid and dashed lines show ±1 and ±1.5 standard deviations of the ensemble spike timing. (B) The normalized fourth moment (excess kurtosis) derived from a moving frame.

The impact of the stimulus input on the density of the ensemble is shown in Figure 7, which shows the spike-timing difference of all neurons in the ensemble with respect to a randomly chosen seed-neuron. The mean spike-timing difference is 0 ms throughout the simulation. This is because the system has complete symmetry, so that all neurons fire, on average, symmetrically before or after any other neuron. However, as evident in Figure 7A, the variance in relative spike-timing decreases dramatically during the stimulus interval. Of note is that the ensemble variance does not simply step down with the onset of the stimulus, but rather dynamically diminishes throughout the presence of the stimulus. When this occurs, the mean-field term continues to increase in amplitude. Figure 7B shows the evolution of the kurtosis (normalized so that a Gaussian distribution has a kurtosis of zero). Prior to the stimulus, and reflecting the weak network coupling, the ensemble has a mesokurtotic (broad) distribution. It increases markedly following the stimulus onset, implying a dynamical evolution towards a leptokurtotic (peaked) distribution. That is, although the parameter values are static, the ensemble mean, variance, and kurtosis evolve dynamically in an inter-related fashion. Hence this system exhibits time-dependent interdependence between its first, second, and fourth moments. This is the sort of coupling (between moments of the ensemble density) that neural mass models do not capture.

**Figure 8:**
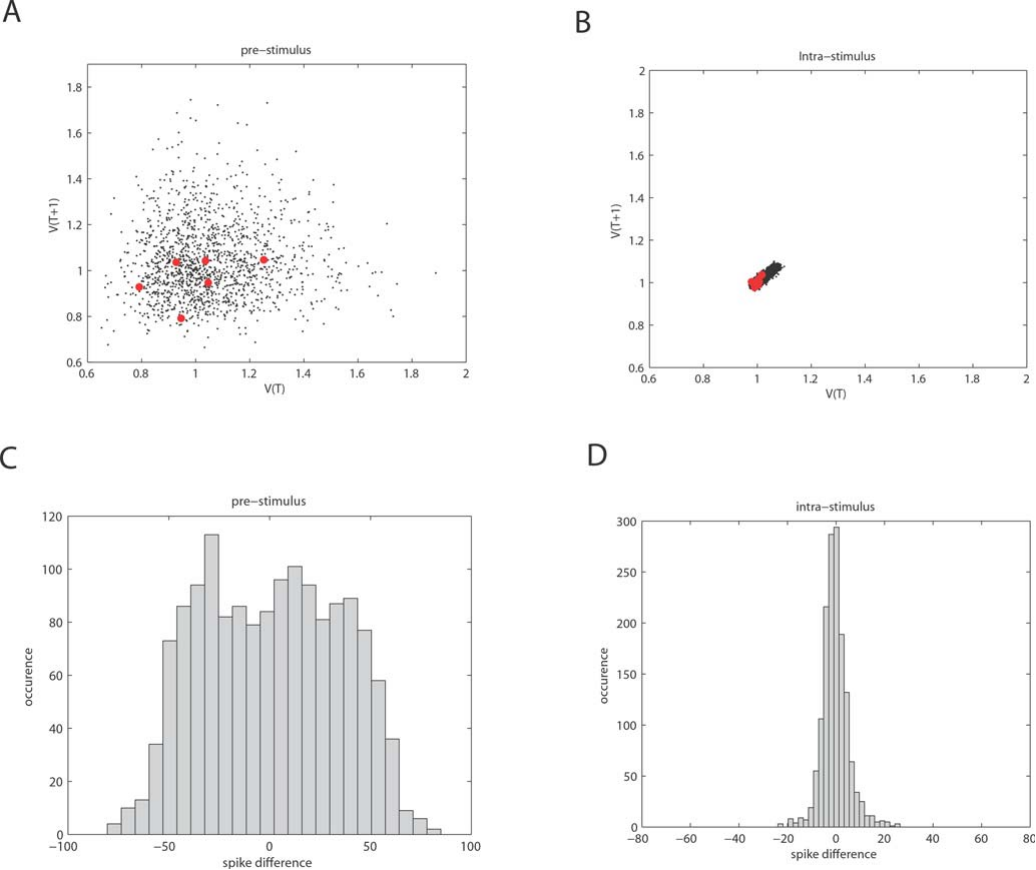
Contraction of spike-timing differences due to synaptic inputs. The left column shows the ensemble state, prior to the stimulus current. The right column shows the intrastimulus activity. Top row: First return map for the cloud interspike delay over five consecutive time steps, before (A) and following (B) synaptic input. The plots are normalized to the average firing rate to control for changes in spike rate. Values for the seed neuron used in Figure 7 are plotted in red. Lower row (C,D): the corresponding spike timing histograms. The ensemble kurtosis increases markedly from sub- to super-Gaussian.

It is important to note that the spatiotemporal structure of the noise remains constant throughout the simulation, as does the intra-ensemble coupling. Hence the appearance of phase locking is an emergent feature of the dynamics and has not been imposed. A dynamic contraction of the ensemble cloud occurs whether the pre-existing noise continues unchanged during the stimulus input—hence increasing the firing rate of each neuron—or diminishes so that, in the absence of coupling, firing rates are kept the same on average. In the latter case (as in Figure 5), there is simply a change from stochastic to periodic firing. The ensemble cloud is visualized directly in Figure 8. The upper row shows the first return map for the ensemble over five consecutive time steps. For each neuron, this is defined as the inter-spike delay at time *t* = *T* plotted against the inter-spike delay for the subsequent spike at *t* = *T*+1. Six such first return state-space values are plotted for all neurons. To control for changes in spike rate, these plots are normalized to the average firing rate. Values for the seed neuron used in Figure 7 are plotted in red. The left column shows the ensemble state, prior to the stimulus current. The right column shows the intra-stimulus activity. The contraction of the ensemble is seen clearly. In addition, the first return map confirms that individual neurons have stochastic dynamics prior to the stimulus, which change to periodic (i.e., a fixed point in the first return map) during the stimulus. The lower row of Figure 7 shows corresponding probability distributions of the inter-neuron spike-timing differences. This reiterates that not only does the distribution contract, but as the mean-field dynamics become strongly nonlinear, the ensemble kurtosis increases markedly from sub- to super-Gaussian.

#### Neural mass dynamics at the mesoscopic scale

Whilst such simulations are illustrative, they are computationally intensive; even when limited to just 250 neurons at <5 s of integration time. As discussed in The Mean-Field Model section, it is possible to study a reduced model representing only the mean ensemble dynamics. This is essentially achieved by generalizing parameter values (such as ion channel thresholds) from individual point values to population likelihood values. Freeman [38] additionally introduced synaptic effects through convolving the inputs with a suitable response kernel as presented in Equation 37. For the simple illustration here, we do not introduce synaptic filtering.

#### *Mesoscopic evolution equations*

For the present purpose, we simulate a single mass with both excitatory and inhibitory neurons [52],[112]. Expected mean states of the ensemble excitatory neurons *μ_e_* = 〈*V_e_*〉 are, as above, based upon Morris Lecar planar dynamics, with slow potassium channel dynamics. Inhibitory neurons, *μ_i_*, respond passively to input from excitatory neurons and feedback to induce additional outward (rectifying) currents in excitatory cells. In the microscopic system considered above, interneuron coupling was via a direct pulse during presynaptic depolarization. At the mesoscopic scale, neuronal coupling is via neural firing pulse densities, *ς_a_*, which capture the expected neuronal firing rate, given the mean neuronal transmembrane potential *ς*(*μ_a_*) for *a* = *e*,*i*. Assuming a Gaussian distribution of individual neuronal firing thresholds, one obtains a symmetric sigmoid-shaped function for *ς_a_* as per The Mean-Field Model section. The dynamics are thus of the form
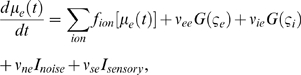
(89)

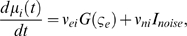
(90)where the function *G* represents the coupling between mean firing rates and induced synaptic currents. By targeting either Na^+^ or Ca^++^ currents and including (postsynaptic) voltage-dependent effects, this function can incorporate, to a first-order approximation, a variable proportion of AMPA or NMDA-like kinetics [52]. The coefficients *ν_αβ_* represent the synaptic density between excitatory (*e*) and inhibitory (*i*) populations or from the stochastic/noise (*n*) or sensory (*s*) inputs. Note that both populations receive stochastic inputs but only the excitatory population receives the sensory input *I_sensory_*. The functions *f_ion_* are the same as for the microscopic system including the slow potassium channel—although they are now parameterized by population-wide estimates.

#### *Mesoscopic dynamics*

**Figure 9:**
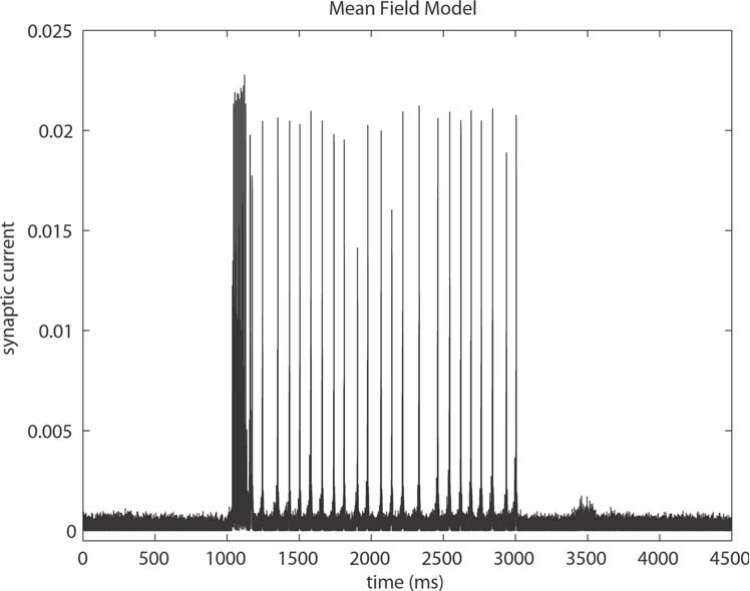
Mesoscopic neural mass model with sensory evoked synaptic currents from *t* = 1,000 ms to t = 3,000 ms. Prior to the stimulus, the system is in a stable fixed point regimen. The stochastic inputs act as perturbations around this point. Although individual neurons exhibit nonlinear dynamics, the ensemble mean dynamics are (linearly) stable to the stochastic inputs until the background current is increased. Then the fixed point state is rendered unstable by the stimulus current and large amplitude oscillations occur. These cease following stimulus termination.

Figure 9 shows the response of a single neural mass to sensory evoked synaptic currents with the same temporal timing as for the microscopic system. Prior to the stimulus, the system is in a stable fixed point regimen. The stochastic inputs act as perturbations around this point, giving the time series a noisy appearance, consistent with the prestimulus microscopic ensemble activity. However, the mechanisms are quite distinct: Individual neurons within the microscopic ensemble fired stochastically, but at uncorrelated times. Hence, at the level of the ensemble, such individual events contribute in a piecemeal fashion. That is, although individual neurons exhibit nonlinear dynamics, the ensemble mean dynamics are (linearly) stable to the stochastic inputs until the background current is increased. In the mesoscopic case, the system as a whole is stable to small perturbations prior to the stimulus current. The temporally uncorrelated stochastic inputs are effectively filtered by the response properties of the system around this fixed point to yield the simulated activity.

In the mesoscopic neural mass, the fixed point state is rendered unstable by the stimulus current and large amplitude oscillations occur. These cease following stimulus termination. This accords with the appearance of stimulus-evoked nonlinear oscillations in the ensemble-averaged response of the microscopic system. In both models, such oscillations abate following stimulus termination. Hence, at a first pass, this neural mass model captures the mean-field response of the microscopic ensemble to a simulated sensory stimulus.

What is lost in the neural mass model? In this model, activity transits quickly from a noise-perturbed fixed point to large amplitude nonlinear oscillations. A brief, rapid periodic transient is evident at the stimulus onset (1,000 ms). The system subsequently remains in the same dynamic state until the stimulus termination. This hence fails to capture some of the cardinal properties of the microscopic ensemble, namely the coupling between the first and second moments (mean and variance). As discussed above, this process underscores the dynamical growth in the mean-field oscillations and the interdependent contraction of the interneuron spike timing variance shown in Figures 6 and 7. Because of this process the system is far more synchronized than prior to the stimulus. This synchronization leads to the damped mean-field oscillations evident in the ensemble system after the stimulus termination (3,200 ms→4,500 ms), because there is a more coherent ensemble-wide response. What is gained in the neural mass model? The addition of a third dimension (i.e., the inhibitory mean activity) to the dynamics enables the expression of chaotic dynamics [52],[112]. Hence the flow terms in the neural mass model contribute to the expression of aperiodic dynamics in addition to the stochastic inputs. This is not possible in the (planar) single neural dynamics of the microscopic system because chaotic dynamics require at least three degrees of freedom. Thus the dimension reduction afforded by the neural mass approximation allows the introduction of more complex intrinsic dynamics, permitting dynamical chaos. Whilst additional dimensions could be added to the microscopic neurons, this would add to an already significant computational burden. The massive reduction in the computational load of the neural mass approximation also allows extension of the spatial scale of the model by an array of neural masses, coupled to form a small patch of cortical tissue. Such a mesoscopic system can be endowed with additional structure, such as hierarchical [108], scale-free [113], multiscale [114], or small world [115] properties. For the present purposes, we couple a single input neural mass, as modeled above, hierarchically to a sheet with internal hyperbolic (i.e., scale-free) coupling. Intersystem coupling is purely excitatory-to-excitatory. Within the sheet, the coupling drops in proportion to spatial separation and is hence scale-free:
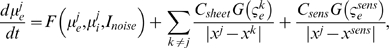
(91)where *F* incorporates all intrasystem dynamics as per Equation 89 and the indices numerate either the sensory node {*sens*} or the nodes within the sheet {*sheet*}. As above, synaptic currents are induced by the pulse density of the presynaptic neurons, rather than directly via individual presynaptic depolarization. The sensory node receives the only direct stimulus-induced currents,
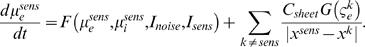
(92)


**Figure 10:**
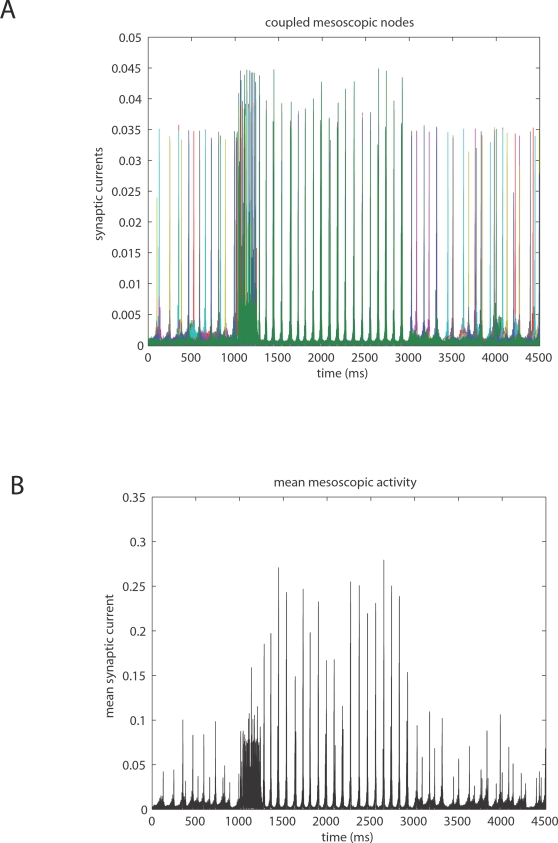
Coupled mesoscopic neural masses with sensory evoked synaptic currents into single sensory node from *t* = 1,000 ms to t = 3,000 ms *C_sens_*>*C_sheet_*. (A) Individual mean synaptic currents of all nonsensory nodes. (B) Total synaptic currents averaged across the nonsensory sheet. Stimulus-evoked activity from the sensory node reorganizes this activity from spatially incoherent to synchronized.

The hierarchical nature of the system is embodied by the targeted nature of the sensory inputs and the separate parameterization of parameters that couple masses to or within the sheet, *C_sens_* and *C_sheet_*, respectively. It would also be possible to increase the degree of forward and backward asymmetry by incorporating purely AMPA-like kinetics for the former and NMDA-like kinetics for the latter, as has been proposed as a mechanism for perceptual inference [116],[117]. Figure 10 shows the response of the system when *C_sens_*>*C_sheet_*. Figure 10A shows the individual mean synaptic currents of all nonsensory nodes. Figure 10B shows the total synaptic currents averaged across the nonsensory sheet. Several features can be noted. For a start, despite use of the same parameters, the coupling of neural masses into an array or sheet leads to the appearance of spontaneous prestimulus activity. Stimulus-evoked activity from the sensory node reorganizes this activity from spatially incoherent to synchronized. Thus the array-averaged synaptic currents increase during the stimulus period. Hence the dynamics at this scale mirror those within the microscopic ensemble, which each node in this simulation is constructed to represent. However, at least in this simulation, the array does not exhibit a dynamic growth in the system-wide currents during the stimulus period. Presumably, if this did occur within the individual mesoscopic nodes, then the array-wide current may also grow dynamically. That is, one would anticipate coupling between moments across ensembles, as discussed in [38].

**Figure 11:**
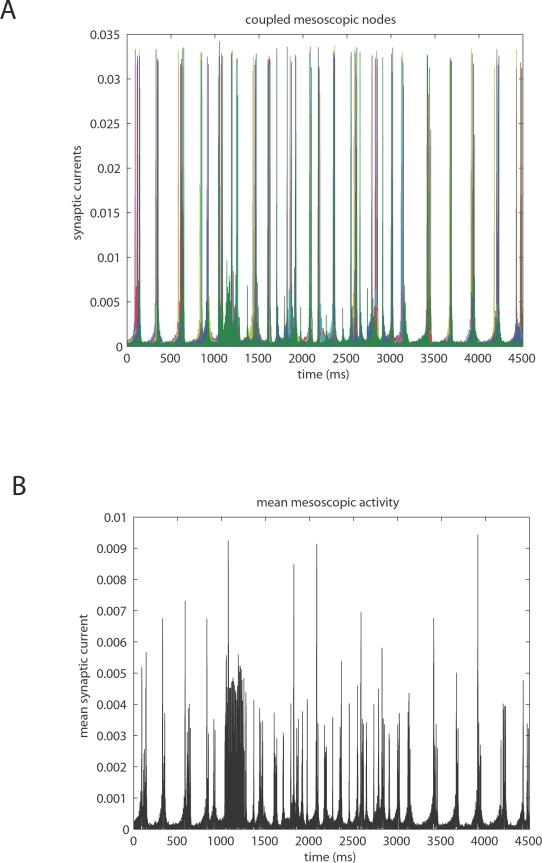
Coupled mesoscopic neural masses with sensory evoked synaptic currents into single sensory node from *t* = 1,000 ms to *t* = 3,000 ms *C_sens_*≈*C_sheet_*. All other parameters as in Figure 10. (A) Individual mean synaptic currents of all nonsensory nodes. (B) Total synaptic currents averaged across the nonsensory sheet. The injection of the externally evoked sensory currents into the prior activity actually has a slightly desynchronizing effect.

Figure 11 shows the simulated activity following an increase in the intrasheet coupling such that *C_sens_*≈*C_sheet_*. All other parameters are unchanged. Spontaneous prestimulus activity is clearly more coherent; consistent with a stronger internally determined dynamical state. The injection of the externally evoked sensory currents into this prior activity actually has a slightly desynchronizing effect, as evident as a decrease in the array-wide average response (Figure 11B). That is, the temporal mismatch between the within-sheet dynamics and the externally induced activity leads to more spatially complex dynamics (an increase in the spatial entropy and hence the information content of the system).

If the stochastic inputs, *I_noise_*, are decreased below a threshold, then the spontaneous activity in the nonsensory array diminishes. The feedback effect of this quiescent activity is to suppress the stimulus-evoked activity in the sensory node. Hence there is a top-down mechanism for the complete suppression of sensory-evoked activity. Presumably, more subtle feedback effects may be possible if more forward versus backward receptor detail was modeled. In summary, these mesoscopic simulations impress a view of sensory-evoked effects as a reorganization of ongoing activity. Depending upon the ratio of internal to sensory-related coupling, this reorganization may lead to an increase or a decrease in the information content of the system dynamics.

#### Neural field dynamics at the whole-brain scale

We now provide brief illustrations of sensory evoked and nonlinear activity as modeled by macroscopic field equations. As discussed in the section entitled Recent Developments in Neural Field Models, these incorporate synaptic filtering and axonal conduction delays, in addition to the population-wide conversion of membrane potentials into firing densities [50]. Significantly, they also permit the incorporation of subcortical systems, such as the thalamus [91]. Recent developments (see the Heterogeneous Connectivity in Neural Fields section) now allow elucidation of the impact of biologically relevant connection heterogeneities on the stability and conduction of cortical activity. The equations, their derivation, and relevant references are provided in the Recent Developments in Neural Field Models section.

#### Macroscopic dynamics

**Figure 12:**
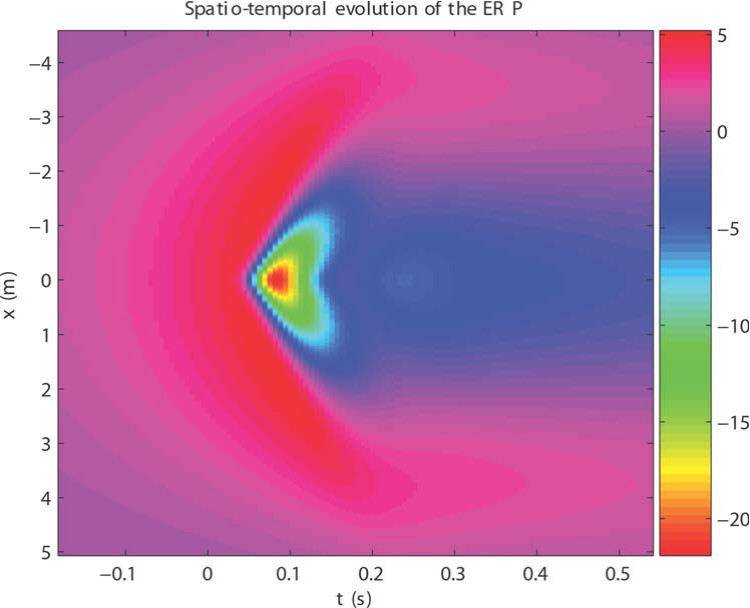
The spatiotemporal evolution of the evoked response of excitatory pulse densities *μ*(*r*,*t*) in an example of sensory-evoked activity (Aquino et. al., unpublished data). The smooth spatiotemporal dispersion of the evoked cortical response and its time delayed corticothalamic volley are evident.

Two crucial differences occur when moving to the macroscopic scale of the corticothalamic field model. Firstly, sensory inputs are modeled as entering the specific nuclei of the thalamus rather than directly into a cortical sensory node. The ensuing evoked corticothalamic activity can then be studied in a biologically informed framework. Second, while prestimulus activity is modeled as a noise-perturbed steady state, the system is not destabilized by sensory inputs. Instead, inputs evoke damped oscillations in the corticothalamic loop [87]. Figure 12 illustrates an example of sensory-evoked activity (Aquino et. al., unpublished data). Evoked afferent pulse densities are shown because they reflect more accurately the expected synaptic currents, through their action on postsynaptic neurons. The smooth spatiotemporal dispersion of the evoked cortical response and its time delayed corticothalamic volley are evident.

#### Summary of numerical simulations

These simulations give insight into the rich neural ensemble dynamics at different spatial scales that arise spontaneously, are evoked by sensory inputs, or follow changes in state parameters. The intention is to demonstrate concrete examples of ensemble dynamics under varying influences of flow and dispersion. The resulting dynamics can be seen to emerge from the interplay of stochastic dispersion and flow-determined ensemble contraction. The view of stimulus-evoked synaptic currents as evoking a bifurcation in neural ensemble activity derives largely from the formative work of Freeman, following detailed physiological studies of the olfactory bulb. One of the key outstanding problems is to reconcile the apparent discrepancy between proposals involving a key role of nonlinear dynamics (see also [118]) and the apparent success of mean-field models to predict measured evoked responses, without recourse to nonlinear dynamics. One approach is to construct a multiscale hierarchy, with self-consistent evolution equations at each scale and to couple the emergent dynamics from fine scales into the activity at coarser scales [114]. Although this permits small scale nonlinear activity to coincide with and influence stochastic macroscopic activity, it requires a somewhat elaborate framework. An alternative approach is to recursively enslave micro- and mesoscopic activity to predicted macroscopic field oscillations by driving them with the predicted mean-field synaptic currents. A problem here concerns the resulting emergence of sustained oscillations within mesoscopic activity and the possible causal inconsistency that this may entail.

The nature and strength of neuronal connectivity varies markedly when considered across the heirarchy of spatial scales. At the microscopic scale, connectivity is dense, concentrated equally in vertical and horizontal directions and, more or less isotropic when considered across different cortical regions. At mesoscopic scales, connectivity has a patchy, colmunar-dominated structure. At macroscopic scales, connectivity is sparser, can be considered exclusively horizontal, and is predominantly excitatory in nature. It is also characterized by heterogenous connections (large fiber tracts) which fulfill functionally defined roles. These rules are reflected in the abstractions and refinements of the models which address the different scales.

## Cognitive and Clinical Applications

In this section, we present three distinct applications of neural ensemble modeling. We first illustrate a computational example, namely decision-making, as implemented in a mean-field model. We then illustrate healthy and pathological activity in neural field models. The healthy example is of the well-known psychophysical phenomenon of auditory streaming—the balance of segmentation versus integration in auditory perception. We then illustrate examples of spatiotemporal dynamics occurring in corticothalamic loops during Absence seizures.

### 

#### Spiking dynamics underlying decision-making

Decision-making is a key brain function of intelligent behavior. A number of neurophysiological experiments on decision-making reveal the neural mechanisms underlying perceptual comparison, by characterising the neuronal correlates of behavior [119]–[121]. In particular, [119]–[124] have studied the neural mechanisms underlying perceptual comparison by measuring single-neuron responses in monkeys trained to compare two mechanical vibrations applied sequentially to the tip of a finger; the subjects have to report which of the two stimuli has the higher frequency. They found neurons in the ventral premotor cortex (VPC) whose firing rate depended only on the difference between the two applied frequencies, the sign of that difference being the determining factor for correct task performance [121]. These neurons reflect the implementation of the perceptual comparison process and may underlie the process of decision-making.

**Figure 13:**
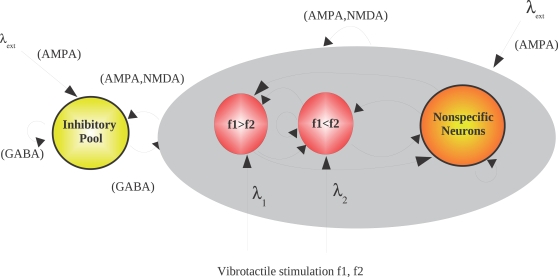
Decision-making neuronal network. Minimal neurodynamical model for a probabilistic decision-making network that performs the comparison of two mechanical vibrations applied sequentially (f1 and f2). The model implements a dynamical competition between different neurons. The network contains excitatory pyramidal cells and inhibitory interneurons. The neurons are fully connected (with synaptic strengths as specified in the text). Neurons are clustered into populations. There are two different types of population: excitatory and inhibitory. There are two subtypes of excitatory population, namely: specific and nonselective. Specific populations encode the result of the comparison process in the two-interval vibrotactile discrimination task, i.e., if f1>f2 or f1<f2. The recurrent arrows indicate recurrent connections between the different neurons in a population.

Figure 13 shows a biophysically realistic computational model for a probabilistic decision-making network that compares two mechanical vibrations applied sequentially (f1 and f2). The model implements a dynamical competition between neurons: The model enables a formal description of the transients (nonstationary) and probabilistic character of behavior (performance) by the explicit use, at the microscopic level, of spiking and synaptic dynamics of one-compartment IF neuron models. The network contains excitatory pyramidal cells and inhibitory interneurons. The excitatory recurrent postsynaptic currents (EPSCs) are mediated by AMPA (fast) and NMDA-glutamate (slow) receptors, whereas external EPSCs imposed on the network are driven by AMPA receptors only. Inhibitory postsynaptic currents (IPSCs) to both excitatory and inhibitory neurons are mediated by GABA receptors. Neurons are clustered into populations. There are two subtypes of excitatory population: namely, specific and nonselective. Specific populations encode the result of the comparison process in the two-interval vibrotactile discrimination task, i.e., if f1>f2 or f1<f2. The neurons in the two specific populations additionally receive external inputs encoding stimulus specific information. They are assumed to originate from the somatosensory area S2 and from the PFC, encoding the frequency of both stimuli f1 (stored) and f2 (present) to be compared during the comparison period, i.e., when the second stimuli is applied (see [125] for details).

The attractors of the network of IF neurons can be studied exhaustively by using the associated reduced mean-field equations. The set of stationary, self-reproducing rates, *ν_i_*, for the different populations, *i*, can be found by solving a set of coupled self-consistency equations. This enables a posteriori selection of parameter regions that contain desired behaviors. In the present case, the essential requirement is that, for the stationary conditions, different attractors are stable. The attractors of interest for our task correspond to the activation (high spiking rates) or inactivation (low spiking rates) of the neurons in the specific populations f1>f2 and f1<f2. The activation of the specific population f1>f2 (f1<f2) and the simultaneous lack of activation of the complementary population f1<f2 (f1>f2), corresponds to an encoding single state associated with a motor response reporting the categorical decision f1>f2 (f1<f2). The lack of activation of both specific populations (spontaneous state) would correspond to an encoding state that cannot lead to a behavioral decision; i.e., there is no answer, or a motor response is generated randomly. The same happens if both specific populations are activated to the same degree (pair state). Because responses in animals are probabilistic in nature, the operating point of the network should be such that both categorical decisions, i.e., both states, are bistable. In addition, we have also shown that the model predicts a behavior consistent with Weber's law if, and only if, the spontaneous state is also a stable state, i.e., when the dynamical operating point of the network is in a regime of multistability. In this way, Weber's law informs the operating point of the network.

**Figure 14:**
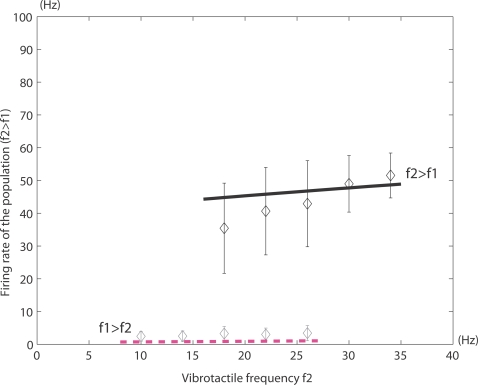
Average firing rate of a neuron as a function of f1 and f2, obtained with the spiking simulations of the response of VPC neurons during the comparison period (to be contrasted with the experimental results shown in Figure 2 of [121]). Diamond points correspond to the average values over 200 trials, and the error bars to the standard deviation. The lines correspond to the mean-field calculations: the black line indicates f1<f2 (f2 = f1+8 Hz) and the red dashed line f1>f2 (f2 = f1−8 Hz). The average firing rate of the population f1<f2 depends only on the sign of f2−f1 and magnitude of the difference, |f2−f1|.

Figure 14 shows numerical simulations corresponding to the response of VPC neurons during the comparison period (to be contrasted with the experimental results shown in Figure 2 of [121]). This figure shows the average firing rate as a function of f1 and f2, obtained with the spiking simulations (diamond points correspond to the average values over 200 trials, and the error bars to the standard deviation). The lines correspond to the mean-field calculations. Black indicates f1<f2 (f2 = f1+8 Hz) and gray indicates f1>f2 (f2 = f1−8 Hz). The average firing rate of the population f1<f2 depends only on the sign of f2−f1 and magnitude of the difference, |f2−f1|; confirming again that Weber's law cannot be encoded in the firing rate, but only in the probability with which that firing rate can be reached (that depends on the sign and magnitude of the difference between f1 and f2).

#### Auditory streaming

One of the applications of neural fields in cognitive processing is found in auditory scene analysis [126], particularly auditory streaming. Intuitively, auditory streaming or stream segregation is like listening to bass and soprano vocalists singing simultaneously. Although the two voices overlap in time, they clearly form two distinct percepts. In the laboratory, a similar effect can be created using sequences of tones. In a typical streaming experiment, two sequences are created using sets of high and low tones. Sequences vary in presentation rate and the frequency difference between the tones. The basic finding (see e.g., [127],[128]) is: (i) when the frequency separation is relatively small and/or the rate is relatively slow, listeners perceive a single integrated melody (or stream) and can accurately report the ordering of the tones, and (ii) when the frequency separation is relatively large and/or the rate relatively fast, people clearly perceive two segregated auditory streams, one with a higher pitch than the other. Essentially, there is a frequency–time boundary (known as the Fission Boundary, FB) beneath which all sequences are heard as integrated, regardless of instructions. There is a frequency–time boundary (known as the Temporal Coherence Boundary, TCB) above which all sequences are heard as segregated, regardless of instructions. In between these two boundaries exists a bistable region in which a sequence can be heard as either integrated or segregated depending upon instructions. Hysteresis phenomena are observed when traversing the bistable regime from either the FB or TCB. Many other auditory phenomena of a related nature are discussed in [128].

To capture the perceptual integration and segregation processes in the human brain, while accommodating contemporary brain theories [1],[2],[4], the authors of [126] proposed a tonotopically organized neural field for peripheral processing with projections to the higher areas that are responsible for cognitive integration. The neural field is tonotopically organized such that the frequency of the acoustic stimulus maps onto a location in neural space. The second nontonotopically organized system may either be represented by a neural field or a subnetwork. Its function is the classification of the peripheral spatiotemporal neural field dynamics. This classification process is not just a measurement (in which case the application of a simple measure to the neural field would suffice) but is itself a dynamic process. In fact, bistability and hysteresis turn out to be properties of the classification process rather than properties of the neural field dynamics. The dynamics of the neural field *μ*(*x*,*t*) are given by the wave Equation 39, which has been extended to accommodate auditory inputs *s*(*x*,*t*) as follows:
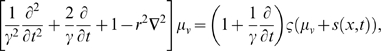
(93)where, as a reminder, *γ* = *c*/*r*, *c* is the speed of spike propagation, and *r* parameterizes the spatial decay of lateral interactions. The external input or stimulus to the neural sheet is *s*(*x*,*t*): **ℜ** ^2^→**ℜ**, which contains all the spatiotemporal characteristics of the auditory input stream. Periodic boundary conditions, *μ*(0,*t*) = *μ*(*L*,*t*), *t*≥0, are used.

**Figure 15:**
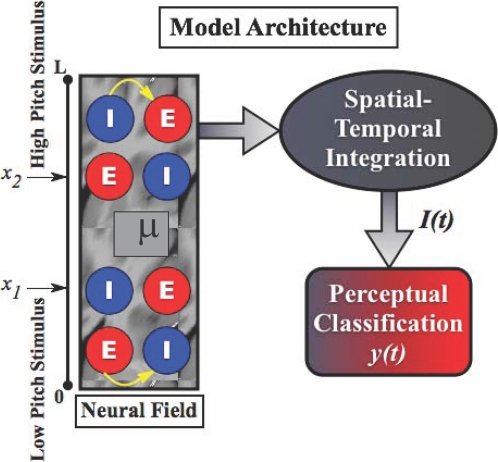
Cortical architecture of the model. The neural field is illustrated by the rectangular box showing the neural activity *μ*(*x*,*t*) composed of inhibitory and excitatory neurons. The input *s*(*x*,*t*) is provided at locations *x_i_* via the Gaussian localization function 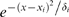 with width 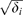. The explicit model parameters used in the simulations are given in [126].

The second network is not tonotopically organized, hence its spatial dimension is of no relevance, when we consider only the competition of two streams. In fact, the ability to show multistable pattern formation is the only relevant property of the network and can be realized in multiple network architectures as discussed in previous sections. A simple multistable subsystem with its scalar state variable *y*(*t*) is given by the equation
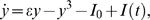
(94)where *ε* is a constant that captures all linear contributions. *I*_0_ contains all constant contributions given rise to the rest state activity. The functional *I*(*t*) is specified as
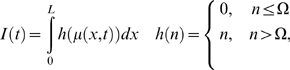
(95)where Ω is a neural activity threshold. Equations 93, 94, and 95 define the dynamics of a stream classification model in one of its simplest forms. Figure 15 illustrates the architecture of the model.

**Figure 16:**
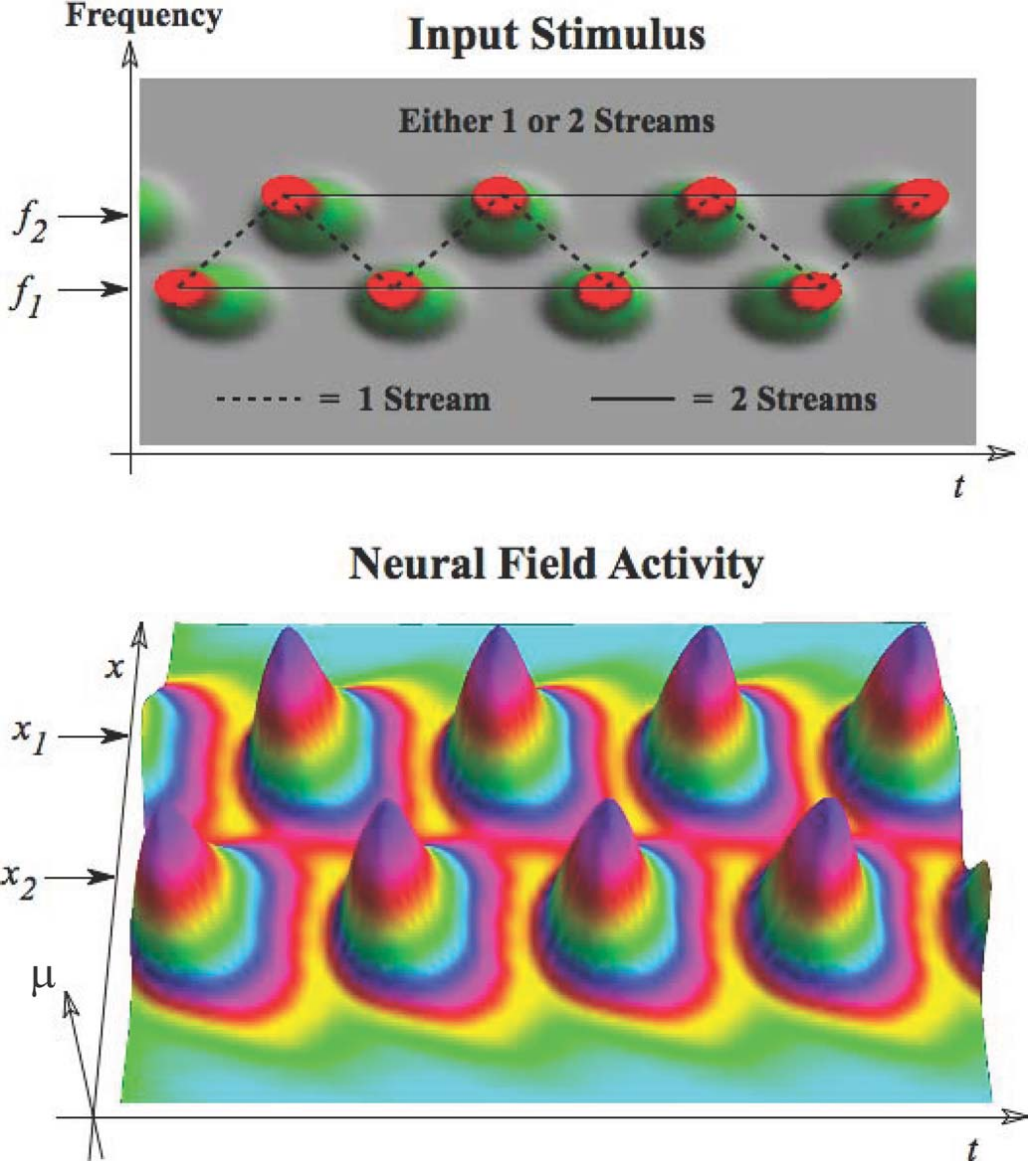
Bistable regime of auditory streaming. The stimulus sequences (top) and its resulting neural field dynamics (bottom).

To understand van Noorden's results, we parametrize a sequence of consecutive tones by their frequency difference, Δ*f*, and their interonset interval, *IOI*. As the neural field evolves, it is integrated across space and time yielding the time-dependent, but scalar, activity, *I*(*t*), driving the second system. *I*(*t*) represents the relevant information from the neural field, *μ*, as a spatiotemporally integrated activity measure, which depends on the amount of dispersion over space and time. The greater the dispersion, the greater will be the value of *I*(*t*) at a given time point. Figure 16 shows the contour lines of neural field activity over space *x* and time *t* for the bistable situation.

**Figure 17:**
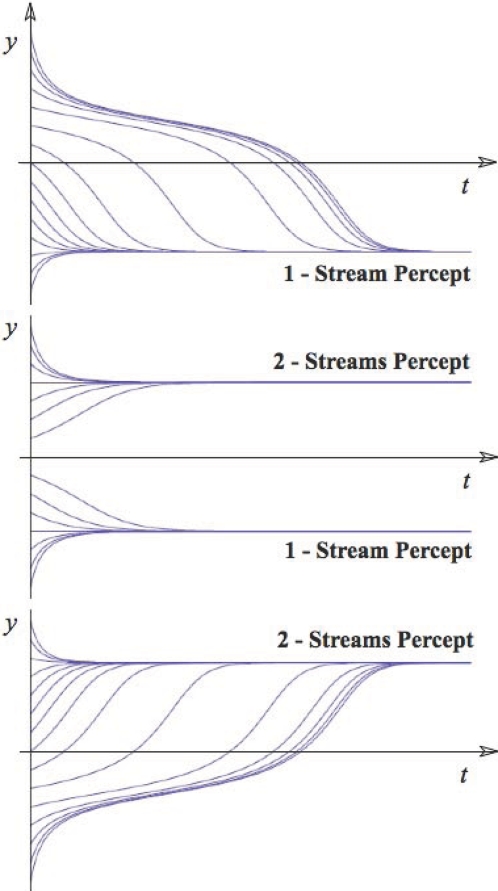
Percept formation. For multiple initial conditions, the time series of *y*(*t*) are plotted for the three regimes, one stream only (top), bistable (middle), and two streams only (bottom).

**Figure 18:**
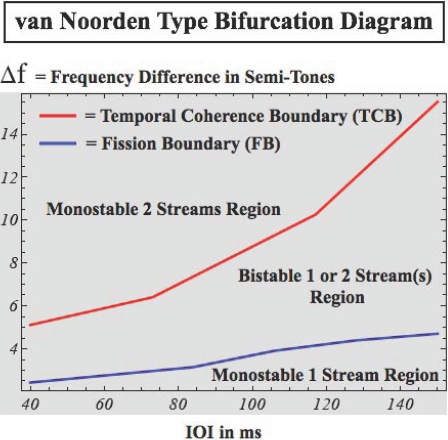
van Noorden's bifurcation diagram. Computational simulations yield the van Noorden's bifurcation diagram as a function of the frequency difference Δ*f* and the *IOI*. The parameter space is partitioned into three regimes, one region with the percept one stream, another region with the percept two streams and a region in between which permits both.

The final state reached by the second system defined in Equation 94 with activity *y* will depend on *I*(*t*) and its own intrinsic dynamics. The curve of the flow is shifted up or down depending on *I*(*t*), creating either one positive or one negative fixed point. For an intermediate value of *I*(*t*), there is a bistable regime in which *y* can assume either one of the fixed points. The negative fixed point is identified with perceiving one stream and the positive fixed point with perceiving two streams. The time series for *y* are shown in Figure 17 for several different initial conditions of the activity *y*. After a transient the activity becomes stationary, displaying three possible scenarios (see Figure 17 from top to bottom): one stream only, or the bistable situation, in which either one integrated stream or two separate streams may be perceived, or finally two streams only. For each choice of Δ*f* and *IOI*, the model Equations 93 and 94 are solved numerically and their stationary states determined. The results are plotted in the 2-D parameter space in Figure 18. TCB and the FB are reproduced in a manner that corresponds nicely to van Noorden's (1975) results including a bistable region [127]. Note that the exact experimental numerical values at which the boundaries occur vary from subject to subject and depend on the experimental methods employed [128].

**Figure 19:**
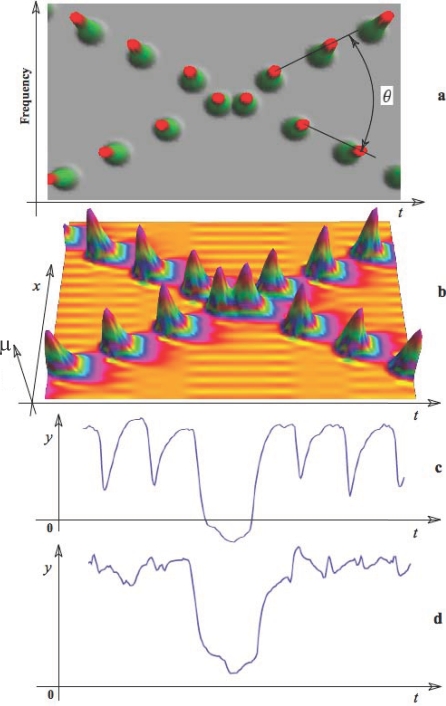
Crossing scales phenomenon. The input tone sequences used to form a percept of crossover are shown in (A). The resulting contours of neural field activity are plotted in (B), together with the final time series of *y*(*t*) shown in (C). In this particular case, the classification system *y*(*t*) does traverse from the positive (two streams) to the negative (one stream) fixed point and back. This trajectory is identified with the percept of crossover. In the case of the bouncing percept, the time series of *y*(*t*) will not cross the x-axis as shown in (D).

We will briefly illustrate another phenomenon. When two interleaved rising and falling tone sequences, as shown in Figure 19, are presented, human subjects report them to be either crossing or bouncing perceptually [128],[129]. This phenomenon is known as the crossing scales phenomenon. The implementation within the neural field model of [126] is straightforward and illustrated in Figure 19.

#### Modeling seizures

**Figure 20:**
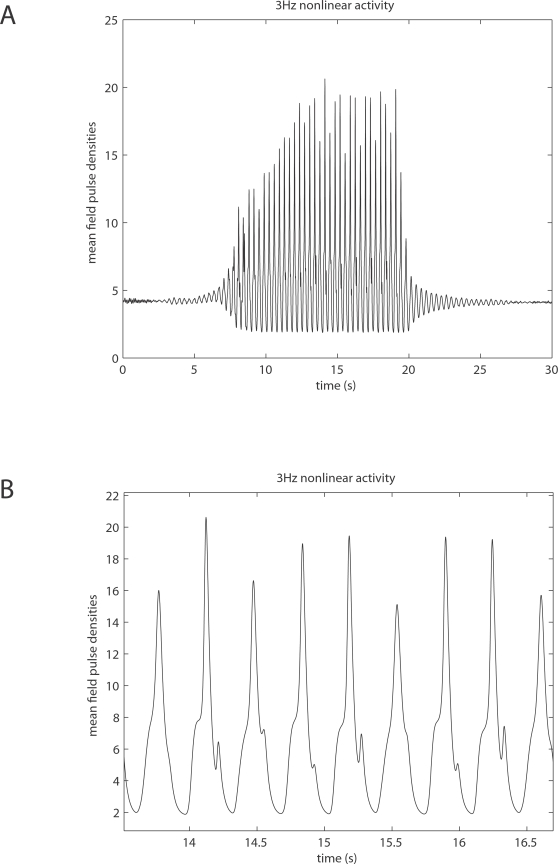
Simulated nonlinear oscillations arising from 3 Hz modal instability in a corticothalamic neural field model. (A) Stochastic activity either side of the seizure can be seen, reflecting the response properties of the stable steady state mode. The large amplitude oscillations arise from a transient change in a corticothalamic state parameter from *t* = 5 s to *t* = 20 s. (B) Shows more detail of the aperiodic oscillations.

Experimental and theoretical arguments propose that the onset of a seizure reflects a bifurcation in cortical activity from damped stochastic activity—where peaks in the power spectrum reflect damped linear resonances—to high amplitude nonlinear oscillations arising from activity on a limit cycle or chaotic attractor [86], [130]–[134]. Figure 20 presents an example of a bifurcation arising from a 3 Hz oscillatory instability in the corticothalamic neural field model of the Recent Developments in Neural Field Models section. Stochastic activity either side of the seizure can be seen, reflecting the response properties of the stable steady state mode. The large amplitude oscillations arise from a transient change in a corticothalamic state parameter from *t* = 5 s to *t* = 20 s. A more systematic analysis of the bifurcations in this neural field model was undertaken in [92]. It was argued that the study of these bifurcations provides a parsimonious explanation of the unique time course, symmetry, onset, and offset of both Absence and tonic clonic seizures, capturing their similarities and the differences. Further analysis of the 3 Hz (Absence) bifurcation in a reduced model argues that interactions between the reticular and specific nuclei of the thalamus contribute importantly to the Absence seizure waveform [135]. In the present simulation, the 3 Hz seizure has inherently aperiodic dynamics, as shown in the right panel of Figure 20.

**Figure 21:**
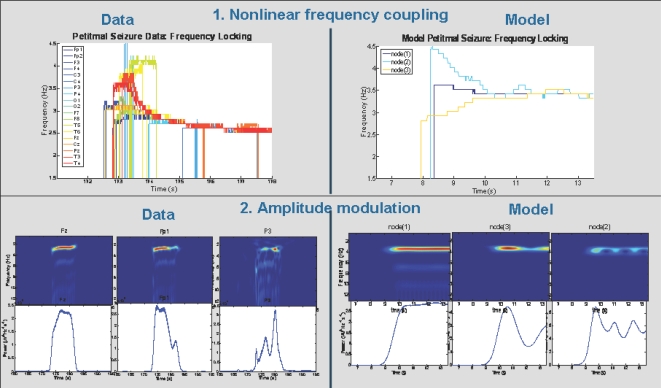
Spatiotemporal activity during 3 Hz spike and wave seizures. (Left) Shows analysis of an Absence seizure in an adolescent male subject. (Right) Shows the results of a simulated seizure in the corticothalamic field model described in the Recent Developments in Neural Field Models section. Top row shows the evolution of the center frequency of the dominant nonlinear mode. Lower row show the amplitude envelope of these modes. Frequencies and amplitudes were derived using a complex Morlet wavelet decomposition of the real and simulated time series.

Neural field formulations, through their implicit treatment of horizontal synaptic coupling, also lend themselves naturally to studying the spatial propagation of seizure activity, a clinically important phenomenon. An analysis of the frequency and amplitude properties of spatially extended 3 Hz seizures (Knock et. al., unpublished data) is presented in Figure 21, comparing data recorded from a young male with Absence epilepsy (left panels) to a simulated seizure in the corticothalamic model (right panels). The top row shows the temporal dynamics of the dominant frequency across a spatially extended array of (real and simulated) electrodes. The observed data (left) shows that the seizure onsets (almost) simultaneously across the scalp, although frontal electrodes lead fractionally. However, onset frequencies range from 2.7 Hz at frontal electrodes to 4 Hz over temporal regions. There follows a pattern of frequency convergence so that within 2 s of seizure onset, all cortical regions express a common frequency of 3 Hz, slowing progressively to 2.5 Hz. The seizure simulated in the corticothalamic model (right) shows a similar pattern. Peak onset frequencies in this model predominantly reflect corticothalamic conduction time lags, which have been parameterized to reflect the varying separation of cortex and thalamus. Subsequent frequency convergence in this model arises from corticortical coupling (there is no intrathalamic coupling in this simulation).

The lower panels show the temporal evolution of the amplitude envelope of activity within the dominant mode. The principal feature of interest in the observed data (left) is the increasing modulation of the amplitude envelope as one moves from frontal electrodes, which have the strongest power, to parietal electrodes, where the onset power is weaker. These differing degrees of amplitude modulation are also present in the simulated seizure (right). Importantly, all parameters of the model are constant during the seizure. Hence the amplitude modulation is due to coupling between nonlinear modes at different spatial locations.

Whereas frequency locking is not surprising in a model with spatial coupling, the amplitude modulation is a novel, emergent property of the nonlinear dynamics.

## Discussion

In conclusion, we have seen that statistical descriptions of neuronal ensembles can be formulated in terms of a Fokker-Planck equation, a functional differential equation prescribing the evolution of a probability density on some phase space. The high dimensionality and complexity of these Fokker-Planck formalisms can be finessed with a mean-field approximation to give nonlinear Fokker-Planck equations, describing the evolution of separable ensembles that are coupled by mean-field effects. By parameterizing the densities in terms of basis functions or probability modes, these partial differential equations can be reduced to coupled differential equations describing their evolution. In the simplest case, we can use a single mode that can be regarded as encoding the location of a probability mass, hence neural mass models. Neural mass models can be generalized to neural field models by making the expectations a function of space, thereby furnishing wave equations that describe the spatiotemporal evolution of expected neuronal states over the cortical surface. We have tried to show the conceptual and mathematical links among the ensuing levels of description and how these models can be used to characterize key dynamical mechanisms in the brain.
